# Another look at the mechanism involving trimeric dUTPases in *Staphylococcus aureus* pathogenicity island induction involves novel players in the party

**DOI:** 10.1093/nar/gkw317

**Published:** 2016-04-25

**Authors:** Elisa Maiques, Nuria Quiles-Puchalt, Jorge Donderis, J. Rafael Ciges-Tomas, Christian Alite, Janine Z. Bowring, Suzanne Humphrey, José R. Penadés, Alberto Marina

**Affiliations:** 1 Instituto de Biomedicina de Valencia (IBV-CSIC) and CIBER de Enfermedades Raras (CIBERER), 46010 Valencia, Spain; 2 Departamento de Ciencias Biomédicas, Facultad de Ciencias de la Salud, Universidad CEU Cardenal Herrera, 46113 Moncada, Valencia, Spain; 3 Institute of Infection, Immunity and Inflammation, College of Medical, Veterinary and Life Sciences, University of Glasgow, Glasgow G12 8TA, UK

## Abstract

We have recently proposed that the trimeric staphylococcal phage encoded dUTPases (Duts) are signaling molecules that act analogously to eukaryotic G-proteins, using dUTP as a second messenger. To perform this regulatory role, the Duts require their characteristic extra motif VI, present in all the staphylococcal phage coded trimeric Duts, as well as the strongly conserved Dut motif V. Recently, however, an alternative model involving Duts in the transfer of the staphylococcal islands (SaPIs) has been suggested, questioning the implication of motifs V and VI. Here, using state-of the-art techniques, we have revisited the proposed models. Our results confirm that the mechanism by which the Duts derepress the SaPI cycle depends on dUTP and involves both motifs V and VI, as we have previously proposed. Surprisingly, the conserved Dut motif IV is also implicated in SaPI derepression. However, and in agreement with the proposed alternative model, the dUTP inhibits rather than inducing the process, as we had initially proposed. In summary, our results clarify, validate and establish the mechanism by which the Duts perform regulatory functions.

## INTRODUCTION

Staphylococcal pathogenicity islands (SaPIs) are mobile genetic elements that carry and disseminate virulence genes in *Staphylococcus aureus* (1–3). They reside passively in the host chromosome under the control of Stl, a global SaPI-encoded repressor. Following infection by a helper phage, or induction of a resident prophage, SaPIs excise, replicate autonomously and are packaged in phage-like particles composed of phage virion proteins (4,5), leading to very high frequencies of inter- and intrageneric transfers (6,7). To initiate the SaPI cycle, a specific phage-encoded protein binds to the SaPI-encoded repressor Stl, acting as an antirepressor (8,9). Both the trimeric and the dimeric phage-encoded Dut proteins are the antirepressor proteins for a subset of SaPIs, including SaPIbov1, SaPIbov5 or SaPIov1 (8–10).

The fact that the trimeric Duts were one of the SaPI inducers aroused our curiosity. Why viruses encode an enzyme already present in their prospective eukaryotic or prokaryotic host cells is an intriguing question. As with our model, in which Duts were involved in the transfer of different SaPIs, others have also proposed that virus-encoded Duts could be moonlighting proteins with different regulatory functions (11). Our laboratories have recently focused on the elucidation of the mechanisms by which Duts perform their regulatory role.

In response to this question, and surprisingly for a metabolic enzyme, a comparison of trimeric Dut sequences from various staphylococcal phages revealed high sequence similarity, except for a nonconserved central region, that we defined as motif VI (8) (Supplementary Figure S1A). This motif is highly divergent among *S. aureus* phage enzymes but, importantly, is not required for enzyme activity (12) and is absent in some functionally related Duts from other species (Supplementary Figure S1B). However, our results analyzing the Dut protein from phage 80α (Dut80α) revealed that motif VI is essential for interaction with the SaPI-encoded Stl repressor, determining the affinity with which the Dut proteins bind to the Stl repressor (8,9).

Interestingly, although motif VI is necessary, it is not sufficient to induce the SaPI cycle. Unexpectedly, the strongly conserved C-terminal P-loop like motif V, present in all characterized trimeric Duts (from phage to human), also plays a key role in mediating derepression. Our crystallographic, mutagenic and *in vivo* analyses suggested that binding to dUTP orders the C-terminal motif V of the phage 80α encoded Dut over the active center, rendering this protein in the conformation required for SaPI derepression (9). Our results also suggested that phage-encoded Duts control both the induction and transfer of SaPIs by a mechanism similar to that reported for eukaryotic G proteins, involving the binding of a nucleotide, dUTP in this case, for partner interaction (9). Bearing in mind the high conservation of motif V, this element is most likely responsible for the ON/OFF mechanism, with the specificity for the target protein provided by the more variable motif VI present in the phage encoded Duts.

Recently however, analyzing a different Dut from phage ϕ11 (Dutϕ11), and using different *in vitro* approaches based on biochemical methods, Szabo *et al*. have proposed an alternative model involving Duts in SaPIbov1 derepression (13). Remarkably, both models involve dUTP as a second messenger, although Szabo *et al*. propose that it is the dUTP free form of the Dutϕ11 which interacts with Stl when the dUTP pool is reduced (13). The authors suggested that the dUTP and Stl compete for binding to the Dutϕ11. However, the structural basis of this competition remains elusive. This alternative model also proposes that the C-terminal motif V is not required to interact with the Stl repressor (13). In addition to this discrepancy, when analyzing *in vitro* the Dut from *Mycobacterium tuberculosis*, which does not contain the extra motif VI but interacts with the SaPIbov1 Stl repressor, the same group also proposed that the motif VI does not have a role either in the formation of the Stl:Dut complex or in removing the SaPIbov1 Stl repressor from its cognate DNA binding site (14). However, in another study using the Dut from phage ϕ11, the same group also suggested that although not involved in the formation of the Stl–Dut complex, the motif VI could be important for the disruption of the Stl–DNA complex (15).

In this paper, using similar approaches to those used by Szabo *et al*., complemented with new structural and biological *in vivo* data, we have revisited our previously proposed model. Our results clearly involve both Dut motifs V and VI in binding to the Stl repressor, and surprisingly, also involve motif IV in the Dut:Stl interaction. Our current results confirm that the motif V competent conformation required for SaPI derepression is not that induced by the binding of the dUTP, as previously proposed. Importantly, however, this domain must be ordered somehow, although the SaPI inducing competent conformation of this domain remains to be elucidated. Our results also support our previously proposed G protein-like mechanism but in an opposite way, where the triphosphate form switches ‘off’ the system,’ confirming dUTP as a second messenger.

## MATERIALS AND METHODS

### Bacterial strains and growth conditions

The bacterial strains used in these studies are listed in Supplementary Table S1. The procedures for preparation and analysis of phage lysates, in addition to transduction and transformation of *S. aureus*, were performed essentially as previously described (16,17).

### DNA methods

General DNA manipulations were performed using standard procedures. The oligonucleotides used in this study are listed in Supplementary Table S2. The labeling of the probes and DNA hybridization were performed according to the protocol supplied with the PCR-DIG DNA-labeling and Chemiluminescent Detection Kit (Roche).

### Plasmid construction

The plasmid constructs expressing the different Dut proteins were reported previously (Supplementary Table S3) or were prepared by cloning PCR products obtained with the oligonucleotide primers listed in Supplementary Table S2. All clones were sequenced by the Institute Core Sequencing facility. Dut proteins were expressed in *S. aureus* under inducing conditions from the P*cad* promoter in the expression vector pCN51, as previously described (8).

The gene encoding the Stl from SaPIbov1 was cloned in the expression vector pETNKI-hisSUMO3-LIC (kindly supplied by Patrick Celie, NKI Protein facility). This vector contains 6His-tag for affinity purification and SUMO protein to increase solubility. The His-SUMO3 tag can be removed using the enzyme SUMO Protease 2 (SENP2). The ligation-independent cloning (LIC) system was used to clone the insert (18). To amplify the *stl* gene the Stl-M1SUMO-FW and Stl-N267SUMO-RV primers (Supplementary Table S2) were used and genomic DNA from *S. aureus* strain JP3603 was used as the template. The resulting vector, pETNKI-Stl, was sequenced for verification at the IBV Core Sequencing Facility.

### Protein expression and purification

The expression of His-tagged *wild-type* (WT) and mutant Dut proteins were done in *E. coli* BL21 (DE3) (Novagen) strain transformed with the corresponding gene cloned in pET-28a plasmid (Novagen) (Supplementary Table S3), as previously described (9). Briefly, proteins were overexpressed by first growing the cells to exponential phase at 37°C in LB medium supplemented with 33 μg/ml kanamycin, followed by the addition of 1 mM isopropyl-β-D thiogalactopyranoside (IPTG) for 3 h to induce protein expression. After the induction, cells were harvested by centrifugation, resuspend in buffer A (100 mM HEPES pH 7.5, 500 mM NaCl) supplemented with 1 mM phenylmethanesulfonyl fluoride (PMSF) and lysed by sonication. The lysate was clarified by centrifugation and the soluble fraction was loaded on a His Trap HP column (GE Healthcare) pre-equilibrated with buffer A. The column was washed with the same buffer supplemented with 10 mM imidazole and proteins were eluted with buffer A supplemented with 500 mM imidazole. The eluted proteins were concentrated and loaded onto a Superdex S200 (GE Healthcare) equilibrated with buffer B (100 mM HEPES pH 7.5, 250 mM NaCl) for size exclusion chromatography. The fractions were analyzed by SDS/PAGE and those fractions showing purest protein were selected, concentrated and stored at −80°C.

For the expression of Stl, the vector pETNKI-Stl was transformed in *E. coli* BL21 (DE3) (Novagen) strain. Cells were grown at 37°C in LB medium supplemented with 33 μg/ml kanamycin up to OD_600_ = 0.5–0.6 and protein expression was induced with 0.1 mM IPTG at 20°C for 16 h. After induction cells were harvested by centrifugation at 4°C for 30 min at 3500 × *g*. The cell pellet was resuspended in buffer STL (400 mM NaCl, 75 mM HEPES pH 7.5, 5 mM MgCl_2_, 2 mM DTT) supplemented with 1 mM PMSF and sonicated. Soluble fraction was obtained after centrifugation at 16 000 × *g* for 40 min, and it was loaded on a pre-equilibrated His Trap HP column (GE Healthcare). After washing with 10 column volumes with buffer STL supplemented with 10 mM imidazole, the protein was eluted with buffer STL supplemented with 300 mM imidazole. The eluted protein was digested for His-SUMO3 tag removal using SENP2 at a molar ratio 1:50 (protease:eluted protein) for 16 h at 4°C and slow shaking. After digestion, the sample was concentrated and loaded on a Superdex S200 (GE Healthcare) pre-equilibrated with buffer STL. Fractions were analyzed by SDS/PAGE and those fractions with purest digested Stl protein were selected, concentrated and stored at -80°C.

### Biolayer interferometry (BLI)

The kinetics parameters of the interaction, binding affinity (*K*_D_) and rate constants of association (*k*_on_) and dissociation (*k*_off_), between Duts and Stl were measured by biolayer interferometry (BLI) using the BLITz system (FortéBio). Proteins were diluted in buffer STL and the assays were carried out in the same buffer, when necessary it was supplemented with the corresponding uracil nucleotide, 0.5 mM of dUPNPP or 5 mM or dUMP. A non-reducing buffer was used to evaluate the interaction of Dut80α^D81A C-C^ with Stl (400 mM NaCl, 75 mM HEPES pH7.5, 5 mM MgCl_2_). Biosensor hydration, baselines and dissociation analysis were carried out in buffer STL without nucleotide addition. For each interaction, the corresponding His-tagged Dut was immobilized on Ni-NTA biosensors (FortéBio) at 1 μM concentration. At least five different dilutions of Stl (from 4 to 0.062 μM plus the reference without Stl) were used in the association and dissociation steps for each Stl:Dut interaction measured, adjusting the highest concentration of Stl to 10 times the estimated *K*_D_ (Table 1). Kinetics values calculation and data analysis were performed with BLItz Pro 1.2 software. A 1:1 model was employed to fit the data.

**Table 1.** Biolayer Interferometry kinetics values of Stl:Duts interaction in the presence or absence of uracil nucleotides

Protein^a^		Nucleotide^b^	*K*_D_ (*k*_off_/*k*_on_) (M)	*k*_on_ (M^−1^s^−1^)	*k*_off_ (s^−1^)
Dut80α	Dut80α^WT^	-	4 × 10^−8^	2.5 × 10^4^ ± 6 × 10^2^	1 × 10^−3^ ± 3 × 10^−5^
		dUPNPP	4.66 × 10^−7^	3 × 10^3^ ± 2 × 10^2^	1.4 × 10^−3^ ± 3 × 10^−5^
		dUMP	2.35 × 10^−8^	3.4 × 10^4^ ± 4 × 10^2^	8 × 10^−4^ ± 2 × 10^−5^
	Dut80α^ΔV^	-	5.3 × 10^−7^	1.7 × 10^4^ ±4 × 10^2^	9 × 10^−3^ ± 2 × 10^−4^
		dUPNPP	5.25 × 10^−7^	8 × 10^3^ ± 1 × 10^2^	4.2 × 10^−3^ ± 7 × 10^−5^
		dUMP	6.02 × 10^−7^	8.3 × 10^3^ ± 2 × 10^2^	5 × 10^−3^ ± 1 × 10^−4^
	Dut80α^D81A^	-	1.61 × 10^−7^	2.6 × 10^4^ ± 9 × 10^2^	4.2 × 10^−3^ ± 2 × 10^−4^
		dUPNPP	NBD^c^		
	Dut80α^Y84I^	-	NBD		
		dUPNPP	NBD		
	Dut80α^F165A^	-	5.81 × 10^−7^	2.7 × 10^4^ ± 2 × 10^2^	1.57 × 10^−2^ ± 2 × 10^−4^
		dUPNPP	NBD		
	Dut80α^G164S^	-	1.56 × 10^−7^	3 × 10^4^ ± 4 × 10^2^	4.7 × 10^−3^ ± 5 × 10^−5^
		dUPNPP	NBD		
	Dut80α^D81A C-C^ (reducing conditions)	-	5.83 × 10^−8^	2.4 × 10^4^ ± 6 × 10^2^	1.4 × 10^−3^ ± 4 × 10^−5^
		dUPNPP	1.92 × 10^−7^	2.6 × 10^4^ ± 8 × 10^2^	5 × 10^−3^ ± 9 × 10^−6^
	Dut80α^D81A C-C^ (non-reducing conditions)^d^	-	6 × 10^−9^	1.4 × 10^5^ ±1 × 10^1^	8.5 × 10^−4^ ± 1 × 10^−5^
		dUPNPP	1.93 × 10^−8^	5.7 × 10^4^ ± 6 × 10^2^	1.1 × 10^−3^ ± 2 × 10^−5^
	Dut80α^ΔVI^	-	NBD		
		dUPNPP	NBD		
	Dut80α ^ΔVI-IV-11^	-	4.56 × 10^−9^	5.7 × 10^4^ ± 3 × 10^2^	2.6 × 10^−4^ ± 6 × 10^−6^
	Dut80α ^IV-11^	-	3.13 × 10^−9^	6.7 × 10^4^ ± 2 × 10^1^	2.1 × 10^−4^ ± 2 × 10^−5^
Dutϕ11	Dutϕ11^WT^	-	1.84 × 10^−9^	5 × 10^4^ ± 3 × 10^2^	9.2 × 10^−5^ ± 3 × 10^−6^
		dUPNPP	3 × 10^−7^	2 × 10^3^ ± 1 × 10^2^	6 × 10^−4^ ± 1 × 10^−5^
		dUMP	1.93 × 10^−9^	3 × 10^4^ ± 2 × 10^2^	5.8 × 10^−5^ ± 7 × 10^−7^
	Dutϕ11^ΔV^	-	1.42 × 10^−9^	2.8 × 10^4^ ± 2 × 10^2^	4 × 10^−5^ ± 2 × 10^−6^
		dUPNPP	3.84 × 10^−9^	1.3 × 10^4^ ± 5 × 10^1^	5 × 10^−5^ ± 1 × 10^−6^
	Dutϕ11^ΔVI^	-	1.14 × 10^−8^	2.1 × 10^4^ ± 1 × 10^2^	2.4 × 10^−4^ ± 3 × 10^−5^
		dUPNPP	1.27 × 10^−8^	1.1 × 10^4^ ± 4 × 10^2^	1.4 × 10^−4^ ± 2 × 10^−6^
	Dutϕ11^ΔV-ΔVI^	-	1.11 × 10^−7^	1.7 × 10^4^ ± 1 × 10^2^	1.9 × 10^−3^ ± 1 × 10^−5^
		dUPNPP	2 × 10^−7^	9.5 × 10^3^ ± 6 × 10^2^	1.9 × 10^−3^ ± 1 × 10^−5^
	Dutϕ11^ΔVI F164A^	-	1.31 × 10^−7^	1.6 × 10^4^ ± 5 × 10^2^	2.1 × 10^−3^ ± 6 × 10^−5^
		dUPNPP	3.78 × 10^−7^	8.2 × 10^3^ ± 6 × 10^2^	3.1 × 10^−3^ ± 2 × 10^−5^
	Dutϕ11^ΔVI-IV-80α^	-	NBD		
	Dutϕ11 ^IV-80α^	-	3.33 × 10^−9^	7.5 × 10^4^ ± 1 × 10^3^	2.5 × 10^−4^ ± 1 × 10^−5^

^a^His(6)-Dut protein purified.

^b^dUPNPP was used at a final concentration of 0.5 mM; dUMP was used at 5 mM.

^c^NBD: not binding detected in the experimental conditions used. *K*_D_ > 1 × 10^−6^M.

^d^Non-reducing buffer: 400 mM NaCl, 75 mM HEPES (pH7,5), 5 mM MgCl_2_.

### dUTPase activity assay

The dUTPase activity was measured by Malachite Green phosphate assay (19,20). This method quantifies the Pi released in 200 μl assay volume of reaction buffer containing 100 mM HEPES pH 7.5, 250 mM NaCl, 5 mM MgCl_2_ and 0.01 U of inorganinc pyrophosphatase (Thermo scientific), and 0.1 μg of the corresponding Dut (21). The reactions were started by addition of dUTP (400μM final concentration) and aliquots were taken at 0, 2, 4, 6, 8 and 10 min and added to 50 μl of malachite green development solution to stop the reaction. After 10 min incubation at room temperature, the Pi production was calculated based on the absorbance at 630 nm and against a previously determined standard curve for Pi. Reactions showed linearity over the time-course of the reaction and the initial velocity was calculated following this procedure using SigmaPlot software.

### Protein crystallization and data collection

Both Dut80α^WT^ and Dut80α^G164S^ mutant proteins were crystallized at 10 mg/ml using sitting drop method in the Crystallogenesis facility of IBV. Dut80α^WT^-dUMP crystals with cubic form were obtained with the Dut80α protein, without any nucleotide previously added, under conditions containing 60% ethanol and 0.1 M NaCl. Dut80α^G164S^ was incubated with 0.5 mM dUPNPP (2-Deoxyuridine-5-[(α,β)-imido]triphosphate; Jena Biosciences) and 5mM MgCl_2_. Dut80α^G164S^ with dUPNPP crystals with cubic form were obtained under 18% ethanol, 0.1 M Tris-HCl and pH 8.5 condition.

Crystals of Dut80α^WT^ were directly frozen in liquid nitrogen without any cryobuffer while Dut80α^G164S^ crystals were frozen in 18% ethanol, 20% glycerol, 0.1 M Tris-HCl and pH 8.5 as cryobuffer for diffraction process. Diffraction data were collected from single crystals at 100 K on ESRF (Grenoble, France), DLS (Didcot, UK) and ALBA (Barcelona, Spain) synchrotrons and processed and reduced with Moslfm (22) and Aimless (23) programs from CCP4 suite (24). The data-collection statistics for the best data sets used in structure determination are shown in Table 2.

### Dut80α^WT^-dUMP and Dut80α^G164S^-dUPNPP structures determination

Both protein structures were solved by molecular replacement with Phaser (30) and an edited Dut80α PDB model (PDB 3zez). Based on previously reported results (9) we excluded from the starting model the high flexibility motif V and the non-conserved motif VI in trimeric Duts (amino acids range 142–170 and 95–127, respectively). This decision was made in order to reduce the imposition of any initial structural conformation to the flexible motif V and the non-conserved motif VI avoiding a possible bias of structural data. Iterative refinement, rebuilding and validation steps were done using programs Coot (25) and Phenix (26). Final models include one Dut molecule (amino acids sequence 2–168 and 2–170) with one dUMP and dUPNPP-Mg bound at the active center for Dut80α^WT^-dUMP and Dut80α^G164S^-dUPNPP, respectively. Both structures had good geometry as indicated by the Ramachandran plots (any residue in the disallowed region). A summary of structures refinement statistics is shown in Table 2.

## RESULTS

### High dUTP concentration blocks Stl:Dut80α interaction

**Figure 1.**
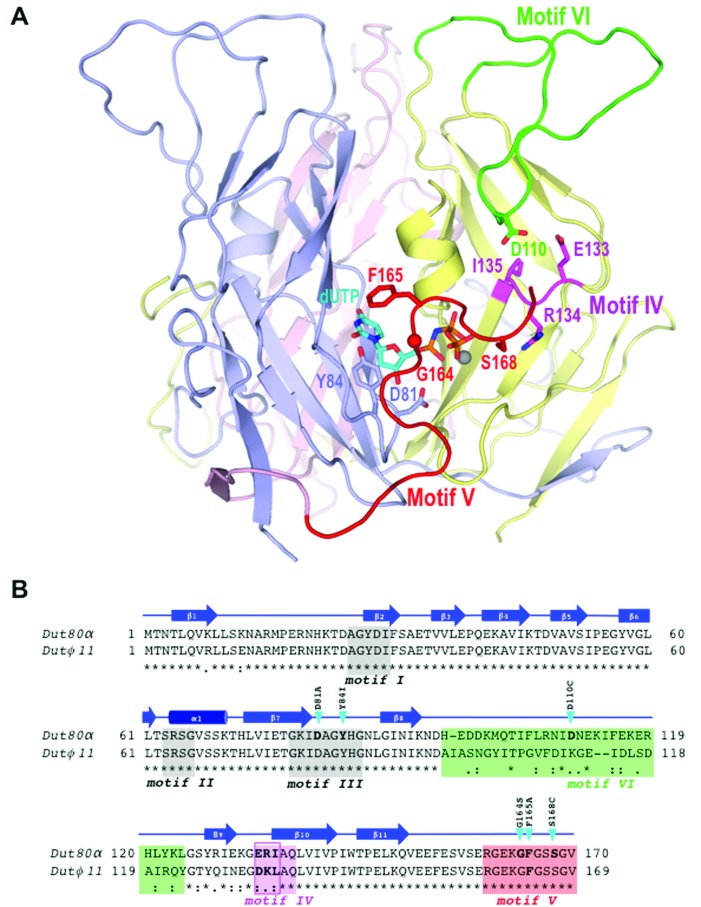
Spatial location in Dut80α^WT^ structure of relevant areas and residues for Dut–Stl interaction analyzed in the manuscript. (**A**) Structure of homotrimeric (monomers in blue, yellow and pink) Dut80α^WT^ bound to dUPNPP-Mg (in sticks with carbon atoms in cyan and gray sphere, respectively; PDB 3ZEZ) highlighting in one monomer the phage Dut specific motif VI (green), the C-terminal conserved motif VI (red) and the exchanged residues from domain IV (magenta). Residues in which mutations are analyzed in the manuscript are shown in sticks and labeled. (**B**) Structure-based sequence alignment of Dut80α and Dutϕ11. The (*) symbol indicates the conserved residues and the (.) or (:) conserved substitutions. The secondary structural elements are shown and labeled above the aminoacid sequence in blue. The five Dut conserved motifs are shaded in gray for motif I, II and III, pink for motif IV and red for motif V, and the *S. aureus* phage specific motif VI in green. The point mutations evaluated in the manuscript and highlighted as sticks in Figure 1A are indicated with cian arrows. For Dut80α^ΔVI^ and Dutϕ11^ΔVI^ motif VI deletional mutants, the highlighted region in green was substituted by Ser-Asn. For Dut80α^ΔV^ and Dutϕ11^ΔV^ C-terminal motif V mutants, a stop codon was introduced at the beginning of motif V (S158* and S157* for Dut80α ^ΔV^ and Dutϕ11^ΔV^, respectively). Motif IV residues exchanged between Dut80α (Glu133-Arg134-Ile135) and Dutϕ11 (Asp132-Lys133-Leu134) are highlighted with a pink square.

As previously mentioned, Szabo *et al*. proposed that the Stl repressor and the dUTP compete against each other to bind to the ϕ11 Dut protein (13). By contrast, our original hypothesis, supported by the analysis of the Dut protein from phage 80α, was that the Stl:Dut interaction occurs once the dUTP has ordered the conserved motif V (Figure 1). To test how the dUTP influences the Stl:Dut80α interaction, we analyzed the binding of Stl to the *wild-type* Dut80α (Dut80α^WT^) in the presence of dUPNPP, a nonhydrolysable dUTP analog, using biophysical and biochemical methods similar to those used by Szabo and co-workers. In agreement with the alternative model proposed by Szabo *et al* (13), the BLI analysis revealed that the presence of the dUPNPP severely affects the formation of the Stl:Dut80α complex (Table 1). The same result was obtained when we analyzed the interaction between Stl and the *wild-type* Dut from phage ϕ11 (Dutϕ11^WT^) by BLI, although Dutϕ11 is more sensitive to the presence of the nucleotide than Dut80α. Thus, the dUPNPP decreased the affinity of the Stl:Dut80α complex by about one order of magnitude, but by two orders for the Stl:Dutϕ11 interaction (Table 1). Importantly, in absence of nucleotide, Dutϕ11^WT^ has 20 times higher affinity for the Stl repressor than Dut80α^WT^ (Table 1), in close agreement with the superior efficacy of the Dutϕ11 in derepressing the SaPIbov1 cycle (8). The differences in affinity for the Stl repressor between both Duts are mainly due to a lower dissociation rate constant for Dutϕ11^WT^ (16-fold lower *k*_off_), showing smaller differences in the association rate (*k*_on_ for Dutϕ11^WT^ only 1.6-fold higher than for Dut80α^WT^). Contrarily, the dUPNPP mainly affects the association step of the Stl:Dut binding, in agreement with the competitive mechanism proposed by Szabo *et al*. (13), without any effect on the Dut80α^WT^ dissociation rate constant and lower impact in the case of Dutϕ11^WT^ (4-fold more impact in *k*_on_ than in *k*_off_) (Table 1).

Since binding to dUTP induces a conformational change dependent on the conserved motif V present in all the trimeric Duts analyzed, two possible scenarios could explain this dUTP-mediated inhibition: i) the motif V is involved, although in an opposite way to what we had previously proposed (with the dUTP-mediated ordering of the motif V blocking the Stl:Dut80α interaction); or ii) as proposed in the alternative model, motif V is not essential in this interaction.

### Motif V implication in SaPI induction and Stl interaction

**Figure 2.**
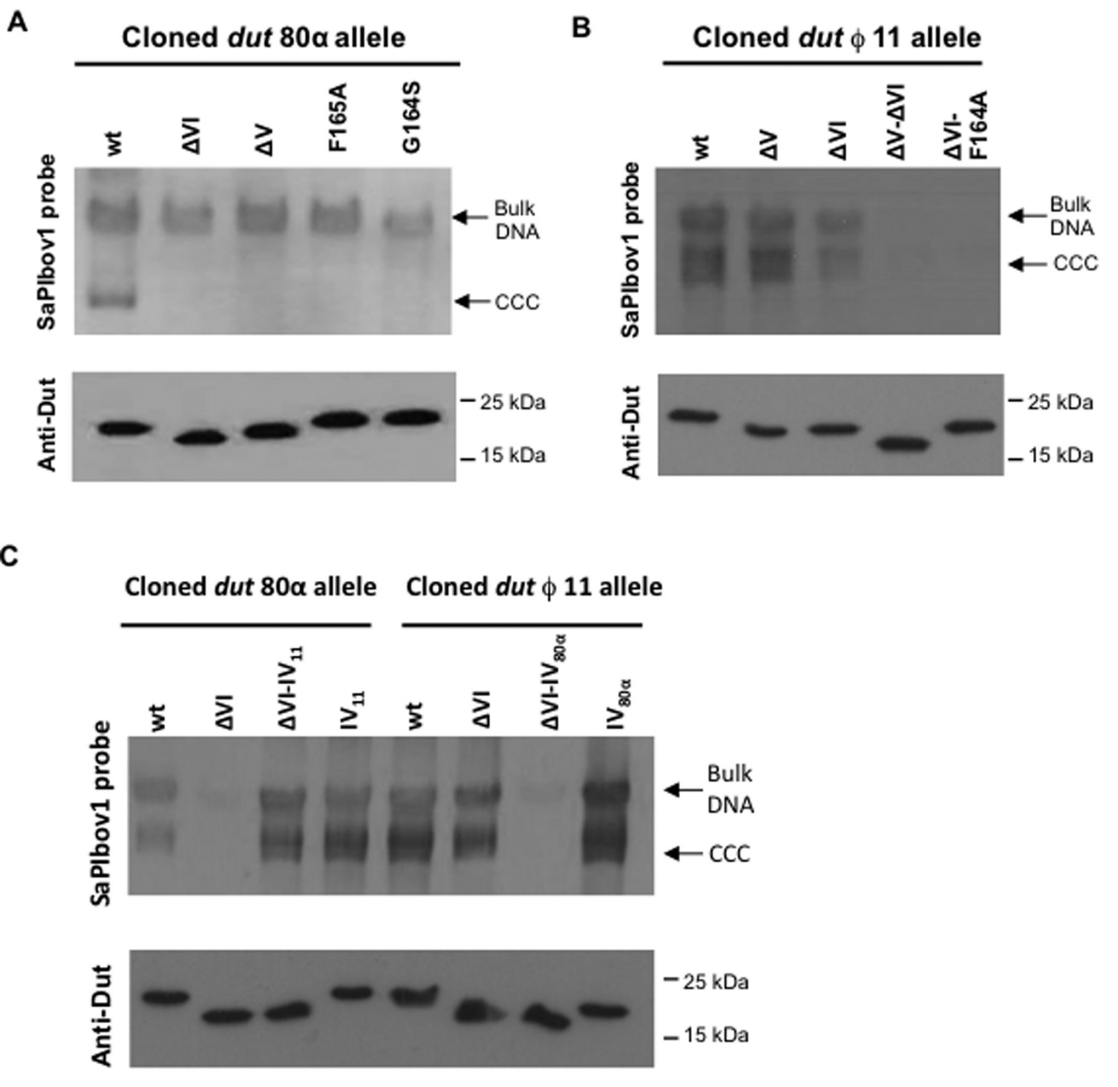
Effects of phage 80α and ϕ11 Dut mutations on SaPIbov1 replication.SaPIbov1 excision and replication after induction of cloned *dut* alleles from phage 80α and ϕ11. A nonlysogenic derivative of strain RN4220 carrying SaPIbov1 was complemented with pCN51 derivative plasmids expressing different 3xFLAG-tagged Dut proteins. One milliliter of each culture (OD540 = 0.2) was collected 2 h after treatment with (**A**) 1 μM CdCl2 (**B**) 0.2 μM CdCl2 or (**C**) 2 μM CdCl2 and used to prepare standard minilysates, which were resolved on a 0.7% agarose gel, Southern blotted and probed for SaPIbov1 DNA. In all the figures (A–C) the upper band is ‘bulk’ DNA; covalently closed circular (CCC) molecules indicate replicating SaPIbov1 DNA. In these experiments, as no helper phage was present, the excised SaPI DNA appears as CCC molecules rather than the linear monomers that are seen following helper phage-mediated induction and packaging. The lower panel is a western blot probed with antibody to the FLAG tag carried by the Dut proteins. (A) phage 80α Dut mutants. wt: Dut80α^WT^; ΔV: Dut80α^ΔV^; ΔVI: Dut80α^ΔVI^; F165A: Dut80α^F165A^; G164S: Dut 80α^G164S^. (B) phage ϕ11 Dut mutants. wt: Dutϕ11^WT^; ΔV: Dutϕ11^ΔV^; ΔVI: Dutϕ11^ΔVI^; ΔV-ΔVI: Dutϕ11^ΔV-ΔVI^; ΔVI F164A: Dut ϕ11 ^ΔVI F164A^. (C) Interchange of motif IV residues between phage ϕ11 and 80α Duts. Cloned *dut* 80α allelle: wt: Dut80α^WT^; ΔVI: Dut80α^ΔVI^; ΔVI-IV11: Dut80α^ΔVI-IV11^; wt-IV11: Dut80α^IV-11^. Cloned *dut* ϕ11 allelle: wt: Dutϕ11^WT^; ΔVI: Dutϕ11^ΔVI^; ΔVI-IV80α: Dutϕ11^ΔVI-IV80α^; wt-IV80α: Dutϕ11^IV-80α^.

To further analyse the Stl:Dut interaction and clearly establish the role of motif V in this process, we made use of the previously characterized Dut80α motif V mutant (Dut80α^ΔV^), in which the C-terminal region of the protein, corresponding to the conserved motif V (Figure 1 and Supplementary Figure S1A), is deleted. This mutant does not induce the island *in vivo* (Figure 2A; (9)) and it binds dUTP (9). Since the dUTP interferes with the formation of the Stl:Dut complex, we investigated if the incapacity of this mutant in inducing the island is because of the presence of the dUTP or because of the deletion in the motif V. For that, the Stl:Dut80α^ΔV^ interaction was analyzed in absence of the dUTP. As shown in Table 1, the Dut80α motif V is essential for Stl:Dut interaction since its deletion decreases affinity for the Stl repressor more than 10 times. Interestingly, dUTP has minimal effect on this mutant, which showed a similar affinity for Stl in the presence or absence of this nucleotide (Table 1), indicating that the Stl-binding inhibition is mediated by the dUTP-induced motif V conformation rather than by the nucleotide itself.

Since these data strengthen the idea that motif V is required for the Stl:Dut interaction, two new possibilities were further analyzed: i) that Stl:Dut interaction requires the *apo* Dut, with a disordered motif V (which, incidentally, is the conformation expected in absence of nucleotide); or more unlikely, ii) that Stl:Dut interaction involves the motif V but in an alternative conformation.

To solve this dichotomy, we initially made use of different Dut80α mutants that cannot order the conserved motif V, even in the presence of the dUTP nucleotide. These correspond to the previously characterized Dut80α D81A (Dut80α^D81A^) and Dut80α Y84I (Dut80α^Y84I^) (9). In addition to these, we also generated and analyzed one additional mutant, F165A (Dut80α^F165A^), with a mutation located in the conserved motif V (Figure 1 and Supplementary Figure S1A). We have previously shown that the catalytic mutants Dut80α^D81A^ and Dut80α^Y84I^ neither induce SaPIbov1 *in vivo*, nor order the motif V in the presence of dUTP, although the Dut80α^D81A^ mutant, but not the Dut80α^Y84I^, binds to dUTP (9). On the other hand, the conserved motif V Phe in position 165 stacks over the uracil ring (Figure 1A) and has been proposed to play a pivotal role both in the ordering of motif V and in the enzymatic activity of the Duts (27,28). As was anticipated, mutation of this residue to Ala (Dut80α^F165A^) yielded a protein without dUTPase activity that was completely unable to induce SaPI derepression (Table 2 and Figure 2A). Importantly, Dut80α^D81A^ and Dut80α^Y84I^ are almost structurally identical to the *apo* form of Dut80α^WT^ protein with motif V completely disordered, despite the presence of the nucleotide in the active center in the case of Dut80α^D81A^ (9). Interaction analyses with Stl, using BLI, showed two different behaviors for these two mutants. Dut80α^D81A^, which is able to bind to the nucleotide, had a slightly lower affinity for the Stl repressor than the Dut80α^WT^ in the absence of the nucleotide, but did not bind in the presence of the dUTP analogue (Table 1). Note that this slight reduction has dramatic consequences *in vivo*, since the Dut80α^D81A^ cannot induce the SaPI cycle (9). By contrast, Dut80α^Y84I^, which showed an even lower (undetectable) affinity for the Stl repressor, was insensitive to the analogue dUPNPP (Table 1), in agreement with the inability of this mutant to bind to the nucleotide (9).

**Table 2.** dUTPase activity

Protein^a^	Activity (μmoles/min/ug protein)^b^
Dut80α^WT^	0.038
Dut80α^G164S^	0.019
Dut80α^F165A^	ND^c^
Dut80α^ΔVI^	0.042
Dut80α ^ΔVI-IV-11^	0.071
Dut80α ^IV-11^	0.038
Dutϕ11^WT^	0.0124
Dutϕ11^ΔV^	ND
Dutϕ11^ΔVI^	0.041
Dutϕ11^ΔV-ΔVI^	ND
Dutϕ11^ΔVI F164A^	ND
Dutϕ11^ΔVI-IV-80α^	0.045
Dutϕ11 ^IV-80α^	0.018

^a^His(6)-Dut protein purified.

^b^Measured as production of PPi at 25°C. 400 μM dUTP. Variation was within ±10%.

^c^ND: no activity detected in the experimental conditions used.

To structurally address these differences we revisited our previously solved structures of Dut80α^D81A^ and Dut80α^Y84I^ (9). We superimposed the Dut80α^D81A^ and Dut80α^Y84I^ structures over the Dut80α structure in complex with the dUPNPP analog. These dockings show that motif V could order, with minimal steric problems, over the active site of Dut80α^D81A^, indicating that this mutation has minimal impact in the active center (Supplementary Figure S2A). By contrast, the Dut80α^Y84I^ mutation induces a twist in the conserved β-hairpin present in motif III (Supplementary Figure S2B), suggesting that the motif V approaching the active center would be structurally hampered.

The Dut80α^F165A^ mutant, affecting motif V, showed an extremely low affinity for Stl even in absence of nucleotide, closely resembling Dut80α^Y84I^ (Table 1). Remarkably, the reduction in affinity for Stl observed in Dut80α^D81A^ and Dut80α^F165A^ mutants is explained by an increment in the dissociation rate constant (*k*_off_) with null or low impact in the association rate constant (*k*_on_) (Table 1). This kinetic mechanism is different to the one observed for the dUTP-induced Stl-binding inhibition, which was achieved by decreasing the association rate (Table 1). Since motif V is always disordered in the two structurally analyzed mutants (Dut80α^D81A^ and Dut80α^Y84I^), these results reinforce the idea that the Stl:Dut interaction requires the *apo* form for Stl binding but with the motif V somehow stabilizing the Stl:Dut complex. This idea was partially confirmed with the analysis of the Dut80α^G164S^ protein, which carries a mutation located in the conserved motif V.

**Figure 3.**
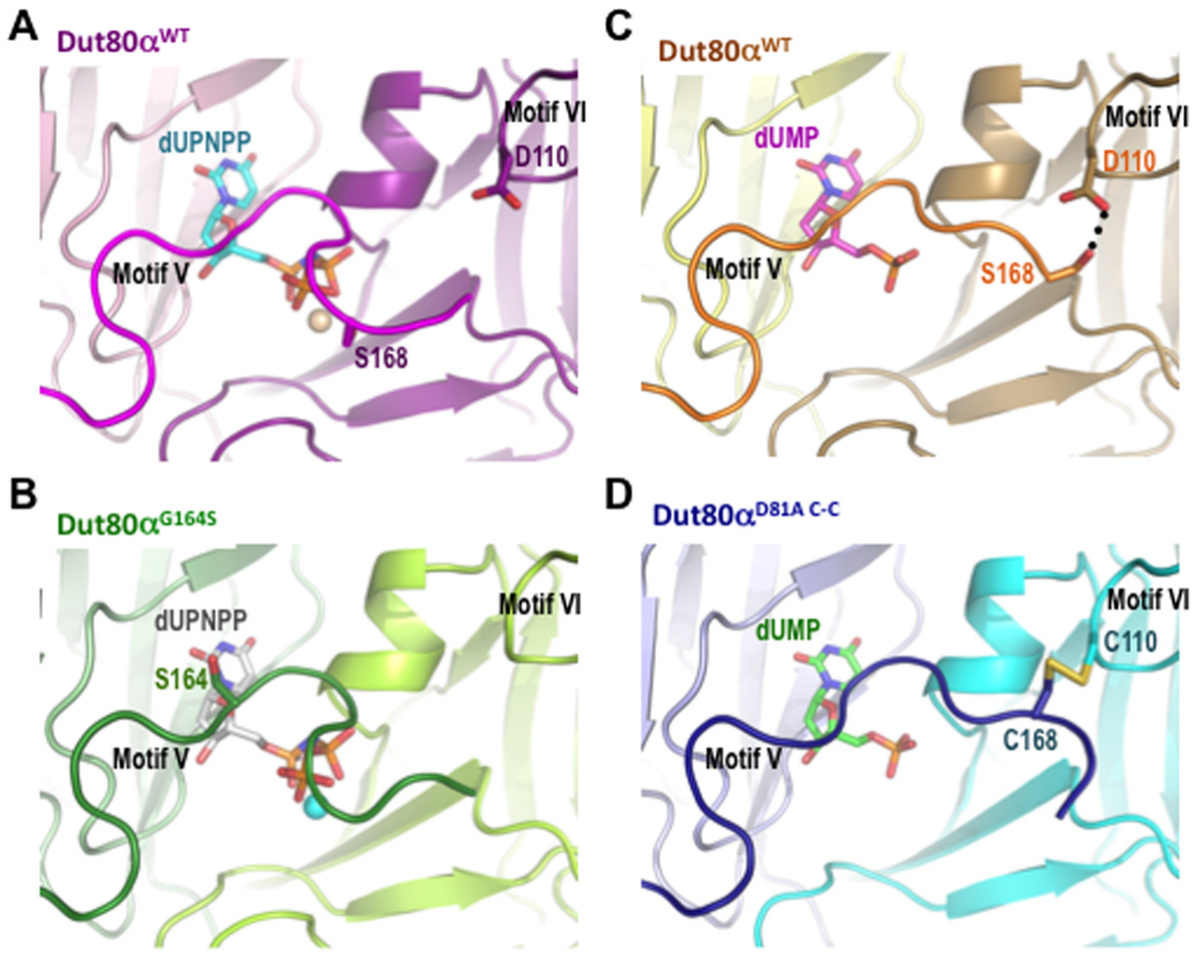
Motif V conformation correlates with the nucleotide bound in the active center. Close view of the active centers of (**A**) Dut80α^WT^ and (**B**) Dut80α^G164S^ bound to dUPNPP, and (**C**) Dut80α^WT^ and (**D**) Dut80α^D81A C-C^ bound to dUMP In the structures, the C-terminal motifs V acquire different conformations depending on which nucleotide is present in the active center. Motifs V and VI are labeled, and the mutated D110 and S168 to Cys forming a disulphide bond in Dut80α^D81A C-C^ are shown in sticks in (A), (C) and (D). The hydrogen bond between these two residues in dUMP bound Dut80α is highlighted by a black dotted line in (C). Mutated residue G164 in Dut80α^G164S^ is shown in sticks in (B). To differentiate the subunits within trimers, each one has been colored with a different shade. Nucleotides are shown in sticks and labeled.

We have shown in a complementary study that the Dut80α^G164S^ mutant has slightly reduced enzymatic activity (Table 2) but is severely affected in its capacity to induce SaPIbov1 (Figure 2A) (29). In view of these data, we speculated that this mutant would be able to order the conserved motif V, although the kinetics of this process would be somewhat affected. As anticipated, the crystallographic characterization of the Dut80α^G164S^ mutant (Table 3) showed that this mutant is able to order the motif V in the presence of dUTP, with an identical conformation to that observed in the Dut80α^WT^ protein (Figure 3A and B). In this conformation, the new Ser side chain is exposed to the solvent on the motif V surface (Figure 3B). No other differences were observed when this mutant was compared with the structures of the previously characterized Dut80α^WT^ (Supplementary Figure S3).

**Table 3.** Crystallographic statistics

	Dut80α^WT^ dUMP	Dut80α^G164S^ dUPNPP
**Processed data**		
Beamline	ID23_1 (ESRF)	XALOC (ALBA)
Wavelength (Å)	0.98	0.98
Space group	P2_1_3	P2_1_3
Cell dimensions		
*a, b, c* (Å)	87.00	87.47
*α, β, γ* (°)	90	90
Resolution (Å)	61.5–2.33 (2.46-2.33)	43.7–2.4 (2.53–2.4)
R_pim_ (%)	0.031 (0.100)	0.029 (0.338)
Mean I/δ(I)	16.2 (6.2)	13.4 (2.0)
Unique reflections	9645 (1387)	8960 (1267)
Completeness (%)	99.9 (100)	99.6 (98.7)
Redundancy	11 (10.6)	5.2 (4.9)
**Refined data**		
Resolution (Å)	61.5–2.33 (2.39-2.33)	43.7–2.4 (2.74–2.4)
R_factor_ (%)	0.201 (0.228)	0.216 (0.263)
R_free_ (%)	0.249 (0.357)	0.249 (0.325)
No. non-hydrogen atoms	1413	1327
RMSD		
Bond deviation (Å)	0.0122	0.002
Angle deviation (°)	1.6326	0.609
Mean B value (Å^2^)	39.426	40.36
Ramachandran Map		
Favored (%)	98.8	97
Allowed (%)	1.2	3
Disallowed region (%)	0	0
PDB accession code	5CCO	5CCT

Values in parentheses correspond to the data for the highest resolution shell.

*R*_pim_ = Σ_hkl_√(1/(*n*-1)) Σ_i_ | I(hkl)_i_ - <I(hkl)> | /Σ_hkl_Σ_i_ I(hkl)_i_.

*R*_factor_ = Σ‖Fo|−|Fc‖/Σ|Fo|.

*R*_free_ is the *R*_factor_ calculated with 5% of the total unique reflections chosen randomly and omitted from refinement.

Interaction analyses with Stl, using BLI, showed that the Dut80α^G164S^, as Dut80α^D81A^, had a reduced affinity for the Stl repressor that is further reduced to undetectable binding levels by the nucleotide (Table 1). Remarkably, the reduction in affinity for Stl observed in the Dut80α^G164S^ mutant, as previously reported for the Dut80α^D81A^ and Dut80α^F165A^ mutants, is explained by an increment in the dissociation rate constant with null or low impact in the association rate constant (Table 1). In summary, these results support our proposed double role for motif V as the switch and recognition element. On one hand, the motif V conformation induced by the dUTP would interfere in Stl binding (dUTP decreases association rate); on the other, an alternative conformation of this motif is implicated in complex stabilization (deletion or mutations affecting this motif increase dissociation rate).

### Role of dUMP in Dut:Stl binding

To explore in more detail this proposed dual function, we analyzed the possibility that motif V should acquire a different conformation in the process of Stl binding and recognition. Szabo and collaborators showed in their alternative model that the substrate dUTP, but not the product dUMP, inhibited the Stl binding to Dutϕ11 (11). As shown in Table 1, and in agreement with the results obtained by Szabo *et al*. with Dutϕ11 (13), the dUMP does not interfere with the formation of the Stl:Dut80α complex. This result was surprising since the alternative model proposed that the dUTP and Stl compete for Dut binding (11) and dUMP and dUTP share the same binding site in the Duts (11). We thought that the differential effect of each nucleotide could be related to the mechanism of Stl binding and we tried to clarify this ambiguity using two complementary strategies: first, we solved the structure of the Dut80α bound to dUMP (Table 3); next, we analyzed the formation of the Stl:Dut complex in the presence of dUMP.

Remarkably, the structure of the Dut80α^WT^ bound to the dUMP showed that motif V was ordered over the active site. Comparison with the structure of Dut80α bound to dUPNPP shows a slight but evident difference in the conformation of motif V, that affects the last four C-terminal residues (Figure 3A and C, and Supplementary Figure S4). This partially conserved region (GSSGV) of the C-terminal motif V (Supplementary Figure S1A), which is involved in *γ*P recognition and enzyme activity (30), adopts an alternative conformation in the presence of dUMP, approaching motif VI. This conformation opens a channel in the active center that could be used for the pyrophosphate product to be released (Figure 3C and Supplementary Figure S5). The reported fast posthydrolysis release of dUTP products, pyrophosphate and dUMP, in Duts (31) would also be favored by the weaker dUMP stabilization of motif V over the active center as is reflected by the B-factors increment of this region (30% higher for motif V than for Dut main body) and the absence of density for the final two residues on dUMP-bound Dut80α. Interestingly, the dUMP-induced motif V conformation was similar to one of our previously characterized Dut80α mutants, in this case the Dut80α D81A D110C S168C (in short Dut80α^D81A C-C^). In this mutant, the D110 (in motif VI) and S168 (in motif V) residues are converted to cysteines (D110C and S168C) (Figure 1). As the crystallographic data confirmed, the proximity of these residues in the native protein allows disulphide bond formation, capturing motif V ordered over the active center with a conformation similar to that obtained with Dut80α^WT^ in the presence of dUMP, with the exception of minimal changes generated by the disulphide bond (Figure 3C and D, and Supplementary Figure S4). Dut80α^D81A C-C^ showed a greater capacity to induce SaPI derepression than Dut80α^WT^, even though it is catalytically inactive (9). In support of our *in vivo* data, the purified Dut80α^D81A C-C^ mutant protein showed an increased affinity for the Stl repressor (Table 1). Importantly, the presence of dUTP had a minor effect on this interaction (Table 1), probably because of the inability of the nucleotide to get access to the active center. Finally, when this mutant protein was analyzed in the presence of reducing agents, it showed a behavior similar to that observed with the Dut80α^WT^ protein (Table 1), suggesting that the dUTP access to the active center should induce conformational changes in motif V that hamper Stl binding. In support of this idea, during the crystallization of the Dut80α^D81A C-C^ protein in oxidized conditions we noticed that the purified protein (from the *E. coli* cells) always contained the dUMP nucleotide in its active center but not the dUTP analog (Figure 3D), even though this mutant was catalytically inactive and dUPNPP was added to the crystallization solution (9).

Since previous results suggested that the competent Dut conformation required for SaPI derepression could be that adopted in the presence of dUMP, we analyzed the effect of this nucleotide on the Dut:Stl interaction. Interestingly, and as previously observed for the dUTP, the dUMP had minimal effect on the Stl binding to the Dut80α^ΔV^ mutant (Table 1), confirming that it is the nucleotide-induced conformation of motif V rather than the presence of nucleotide by itself in the active center that is responsible for the differences observed in Stl binding affinities. Since we proposed that the dUMP could favor an alternative competent conformation of motif V, we initially expected that dUMP-bound Dut affinity for Stl would even be increased in presence of this nucleotide. However, this was not the case. The observed changes in the kinetic parameters were not as we expected, and the dUMP did not induce a clear improvement in the affinity of the Dut80α for Stl (Table 1).

In summary, while the crystallographic data show that dUMP induces conformational changes in the C-terminal part of motif V, the affinity of the Dut:Stl complex is not increased in the presence of this nucleotide, suggesting that this change neither favors nor hampers the interaction with the repressor. Although the motif V is clearly involved in the Stl:Dut80α interaction, more experiments are required to clearly establish the competent conformation of this motif V in derepressing the SaPIbov1 cycle. This is currently under study.

### Motif VI is essential for Stl recognition

In previous work we had demonstrated the importance of Motif VI using complementary strategies, including analysis of the Dut protein from *Staphylococcus epidermidis* phage PH15, which is similar to Dut80α except that it lacks the extra motif VI and, consequently, is incapable of inducing the SaPI cycle (Supplementary Figure S1B) (8). However, based on the suggestions by the Vertessy group that this could not be the case (14), we decided to obtain more clear evidence about the importance of the extra motif VI by analyzing the Stl affinity for Dut80α lacking this extra domain. We substituted this domain (residues 96–124) by two residues (Ser–Asn) and produced the mutant Dut80α^ΔVI^ with intact enzymatic activity (Table 2, Figure 1A and Supplementary Figure S1A). A similar construction of Dutϕ11 lacking the motif VI has been reported previously with only slight differences in its kinetic constants when compared with the *wild-type* enzyme (12). Therefore, it seems that the phage-specific motif VI has no major implications in dUTPase enzymatic activity. In agreement with the proposed implication of motif VI in Stl binding specificity, the Dut80α^ΔVI^ mutant did not show any detectable binding to the Stl repressor, both in the presence and absence of nucleotide, when checked *in vitro* using BLI (Table 1). *In vivo* data confirmed that this mutant is incapable of inducing SaPIbov1, even when overexpressed from a plasmid (Figure 2A), confirming our previous *in vivo* results implicating this domain as having a key role in Stl recognition (8).

### Motif V and VI are also involved in Dutϕ11–Stl interaction

In contrast to these results using the phage 80α Dut, reported experiments seem to indicate that Dutϕ11 does not require either motifs V or VI to interact with Stl (13). Since the Dut proteins encoded by phages 80α and ϕ11 have a completely divergent sequence in their respective motifs VI (Supplementary Figure S1A), but both can induce the SaPIbov1 cycle (8), this opened the possibility that these proteins interact with Stl in a completely different way. Additionally, it should be noted that, as previously indicated, the affinity for the Stl repressor is one order of magnitude higher in Dutϕ11, compared with that from phage Dut80α (Table 1).

To analyze this possibility, we checked the effect of motif V and motif VI deletions in Dutϕ11 both *in vitro* and *in vivo*. Motif V deletion in Dutϕ11 generates a protein (Dutϕ11^ΔV^) with full capacity to induce SaPIbov1, with an affinity for Stl identical to that observed for the Dutϕ11^WT^, but no catalytic activity (Figure 2B and Tables 1 and 2). However, as was observed for Dut80α^ΔV^, the affinity of this mutant for Stl is almost insensitive to dUTP (Table 1), supporting our proposed central role of motif V in dUTP-mediated Stl binding inhibition. To check the role of motif VI in Dutϕ11, we replaced residues 96–123 with Ser-Asn and produced the mutant Dutϕ11^ΔVI^ (Figure 1). This mutant displays superior enzymatic activity to that of the *wild-type* version but a slightly reduced capacity to induce SaPIbov1 (Table 2 and Figure 2B). In line with the *in vivo* data, Dutϕ11^ΔVI^ reduced its affinity for Stl one order of magnitude when checked *in vitro* using BLI (Table 1).

These results were surprising, raising several interesting possibilities. On one hand, it could be possible that the Dut80α and Dutϕ11 have different ways of interacting with the Stl repressor. Another possibility could be that the motifs involved in the Stl binding were identical in both Duts, although the relevance of these motifs in the interaction with the repressor were different, depending on the Dut. Finally, it could also be possible that an additional divergent motif was involved in the Dut:Stl interaction. To solve this interesting mystery, and to clearly decipher the roles of motif V and VI in the Dutϕ11:Stl interaction, we decided to eliminate both domains (V and VI) from the Dutϕ11 protein, generating the Dutϕ11^ΔV-ΔVI^ mutant. In support of the involvement of these motifs in the interaction with the Stl repressor, the double mutant Dutϕ11^ΔV-ΔVI^ showed a significant reduction in its capacity to induce SaPIbov1 derepression and stronger impairment (two order of magnitude *K*_D_ reduction) of Stl affinity than each of the single mutants (Table 1 and Figure 2B), suggesting a synergistic performance of both motifs in the binding to Stl. Furthermore, when the conserved Phe in motif V was mutated to Ala in the Dutϕ11^ΔVI^ mutant, we obtained a catalytically inactive protein mutant, Dutϕ11^ΔVI F164A^, with similar behavior both *in vitro* and *in vivo* to Dutϕ11^ΔV-ΔVI^ (Tables 1 and 2, Figure 2B). Remarkably and as previously shown in the characterization of the Dut80α protein, motif V deletion as much as Phe164Ala mutation renders proteins with almost identical capacity for Stl binding and SaPI derepresion. Altogether, our parallel analysis of Dut80α and Dutϕ11 support a Stl–Dut interaction mechanism that involves motifs V and VI and it seems to be conserved for *S. aureus* phage Duts, although, as will be discussed later, the relevance of the different motifs in the binding to the Stl repressor differs depending on the Dut under study. Finally, the different abilities of Dutϕ11^ΔVI^ and Dut80α^ΔVI^ in binding Stl also suggests the existence of an extra domain involved in the Dut:Stl interaction.

### Motif IV is also implicated in Stl binding

Despite the conserved mechanism of Stl–Dut interaction supported by our data, the difference in affinity for Stl between the motif VI-defective mutants of Dut80α and Dutϕ11 was puzzling. In *S. aureus*, the phage coded trimeric Duts are highly conserved in sequence except in the divergent motif VI (Supplementary Figure S1A). Based on these differences in sequence and in the affinities that the ϕ11 and 80α Duts have for the Stl repressor, we initially assumed that the different SaPIbov1 inducing capacities were exclusively dependent on the divergent motif VI. However, when compared, the sequences of the Dut80α^ΔVI^ and Dutϕ11^ΔVI^ mutants are basically identical, except for nine residues (Supplementary Figure S1A). Surprisingly, the Dut80α^ΔVI^ and Dutϕ11^ΔVI^ mutants have a significant difference in their capacities to induce the SaPIbov1 cycle (Figure 2), suggesting that these differences can also have an important impact controlling the Dut:Stl interaction. We hypothesized initially that three out of the nine different residues (Glu133/Arg134/Ile135 in Dut80α, or Asp132/Lys133/Leu134 in Dutϕ11) could be key elements in Stl recognition since: i) these residues map in the region where the Stl binding motifs V and VI approaches, ii) they are part of the conserved motif IV implicated in Magnesium ion and nucleotide phosphates coordination and iii) the pyrophosphate moiety, which distinguish dUTP from dUMP, is in the close vicinity of these residues (Figure 1A) (32).

To test our hypothesis we interchanged these three residues between the Dut80α^ΔVI^ and Dutϕ11^ΔVI^ mutant proteins, generating mutants Dut80α^ΔVI-IV-11^ and Dutϕ11^ΔVI-IV-80α^, respectively. Since motif VI is extremely divergent and is clearly involved in the Dut:Stl interaction, we initially analyzed the impact of the motif IV residues in the deletion motif VI mutants. Dut80α^ΔVI-IV-11^ and Dutϕ11^ΔVI-IV-80α^ mutants showed intact catalytic dUTPase activities, indicating that the three residue interchange has minor kinetic influence (Table 2). Remarkably, when the three residues comprising the divergent region IV were exchanged, the SaPIbov1 induction efficiency was transferred along with the exchanged amino acids (Figure 2C). The possibility that differential expression of the two genes was responsible for the difference was ruled out by a western blot analysis (Figure 2C), which confirmed that the two genes were expressed at the same levels. With the BLI experiments we confirmed the *in vivo* observations, showing that the affinity for Stl was also transferred along with the residues. The *K*_D_ value of Dut80α^ΔVI-IV-11^ was similar to the one observed for Dutϕ11^WT^, and as was observed for Dut80α^ΔVI^ no Stl complex formation was detected for Dutϕ11^ΔVI-IV-80α^ (Table 1).

Next, we analyzed the impact of the aforementioned three residues in the affinity of the *wild-type* Dut proteins for the Stl repressor. To do this, we exchanged the three residues between the *wild-type* Dut80α and Dutϕ11 proteins, generating the Dut80α^IV-11^ and Dutϕ11^IV-80α^ mutant proteins, respectively, which have intact dUTPase activities (Table 2). However, the interchange has a strong impact on the affinity of the Dut80α for Stl. In this mutant, the affinity for the Stl was increased by one order of magnitude, showing a similar *K*_D_ value than the Dutϕ11^WT^ (Table 1). In agreement with this increase in affinity, Dut80α^IV-11^ displayed a higher capacity to derepress SaPIbov1 *in vivo* when it was expressed from a plasmid (Figure 2C). On the other hand, and in support of the finding that the motif VI present in the Dutϕ11 protein confers to the Dut an increased affinity for the Stl repressor than that present in Dut80α, the Dutϕ11^IV-80α^ mutant showed only a small reduction in its affinity for Stl and almost *wild-type* SaPI induction capacity (Figure 2C). In summary, these results support the involvement of the motif IV in Stl recognition. As previously mentioned, motif IV localizes at the convergence of motifs V and VI, which explains the synergistic participation of all of these motifs in the Stl interaction, and consequently, in the SaPI derepression process.

### The dUTP level does not influence SaPI transfer

It is an interesting mystery how *S. aureus* regulates the dUTP pool. Szabo *et al*. assumed that the intracellular dUTP level would be high in this bacterium, due to the lack of a genomic dUTPase (13). In this scenario, Szabo *et al*. proposed that the role of the phage coded Dut would be to reduce the dUTP pool to physiological concentrations, preventing dUTP incorporation during SaPI replication, which in turn would affect the SaPI cycle. Note, however, that this assumption contradicts previously published literature. It has been shown that high levels of dUTP as a consequence of *dut* deletions or inactivating mutations are detrimental for cell viability, arriving to be lethal for either prokaryotic or eukaryotic cells (33–36).This also suggests that *S. aureus* probably has unexplored pathways to control the dUTP pool, which are completely independent of the phage coded Duts. In support of this idea, the existence of virulent non-lysogenic *S. aureus* strains lacking functional phages, with an extremely well conserved core genome and capability for producing epidemics in rabbits, has recently been reported (37).

Since the previous discussion is completely speculative, and because we cannot measure the dUTP level *in vivo*, we analyzed experimentally whether the absence of the phage coded *dut* has negative effects on SaPI transfer. Our original experiments identifying the SaPI inducers suggested that this is not the case. Using phage 80α that encodes the derepressing proteins for SaPIbov1 (Dut), SaPI1 (Sri) and SaPIbov2 (ORF15), we demonstrated that the 80α *dut* mutant was unaffected in its capacity to induce and transfer the SaPI1 and SaPI2 islands (8). Since the strains used in this study do not encode *dut*, and assuming that the phage coded *dut* were essential for dUTP detoxification, these results rule out the possibility that uracylation is detrimental for the SaPI cycle.

**Figure 4.**
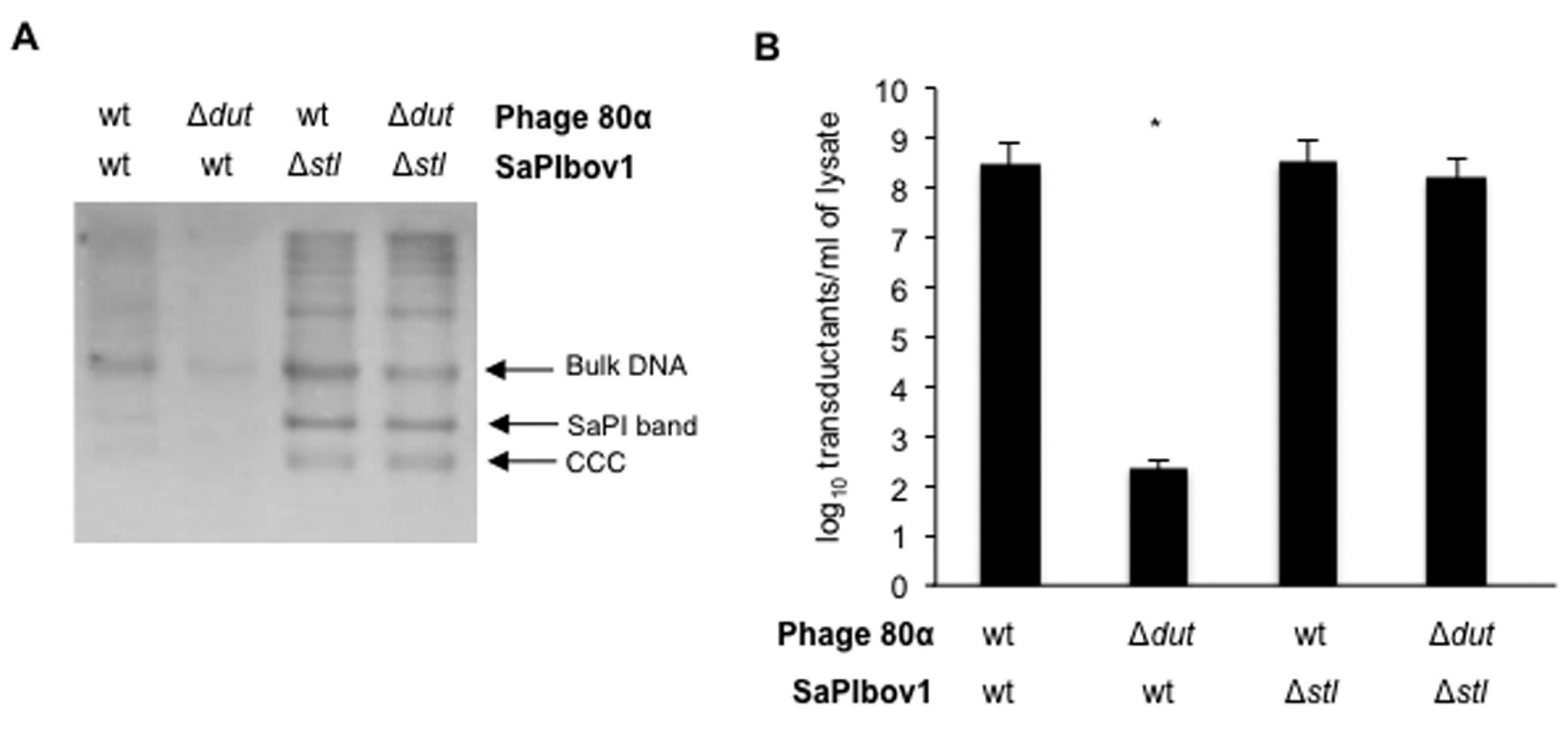
Effect of *stl* mutation in SaPIbov1 replication and transfer. (**A**) Southern blot after induction of 80α wt or 80α Δ*dut* mutant prophages. Samples were isolated 60 min after induction with mitomycin C of the different lysogenic strains carrying SaPIbov1 *tst*::*tet*M (wt) or SaPIbov1 Δ*stl tst*::*tet*M (Δ*stl*). The samples were separated on an agarose gel and blotted with a SaPIbov1-specific probe. The upper band is ‘bulk’ DNA, including chromosomal, phage and replicating SaPI. The intermediate band is SaPI linear monomers released from phage heads. The lower band corresponds to the covalently closed circular (CCC) SaPIbov1 molecules. (**B**) The figure shows the number of transductants (log_10_) per milliliter of induced culture, using RN4220 as recipient strain. The means of results from three independent experiments are presented. The frequency observed in the Δ*dut* mutant is typical of transfer by generalized transduction and is not SaPI specific. Yates' chi-squared test was used to compute *P* values for between-group comparisons; differences that are statistically significant are indicated by an asterisk (*P* < 0.001).

To complete this study, and to analyse how the absence of *dut* influences SaPIbov1 transfer, we made use of a SaPIbov1 mutant in the *stl* repressor. This mutant is constitutively induced in the absence of any inducing phage (16). This mutant was transferred to strains RN10359 (carrying *wild-type* phage 80α) and JP6032 (RN10359 derivative mutant in 80α *dut*). The prophages from both strains were induced using mitomycin C, and the transfer of the SaPIbov1 *stl* mutant analyzed. No differences in SaPI replication or SaPI transfer of the *stl* mutant were observed in the phage *dut* mutant (Figure 4), confirming the previous results that discard a role for uracilation in the SaPI cycle.

## DISCUSSION

Our previous structural and functional analysis of SaPI cycle derepression by the phage 80α Dut indicated that the phage-encoded Duts are signaling proteins with a G-like mechanism of control. Since G-proteins and Duts have different kinetic properties, our proposed conceptual similarity was based on the fact that: i) both protein families work as signaling devices using a nucleotide as a second messenger, ii) P-loop(s) (two in the case of G-proteins) covering the active center are involved in binding of the target protein and iii) hydrolysis of the nucleotide switches both proteins between the ‘on’ and ‘off’ states. Since our original data showed a strong correlation between SaPI derepression and the order of the P-loop motif V, as was observed in the presence of dUTP, our conceptual analogy with the G-proteins also involved the triphosphate nucleotide dUTP as responsible for the ‘on’ state, while the hydrolyzed nucleotide dUMP would turn ‘off’ this signaling cascade. However, the biophysical analysis carried out by Szabo and co-workers, when working with the phage ϕ11 Dut, showed that dUTP precludes the Stl binding to Duts (13). Based on this observation the authors proposed an alternative model by which the Dut proteins induce the SaPI cycle. This alternative model, although provocative and interesting, assumed a high intracellular dUTP level for *S. aureus* in basal growth conditions which has been shown to be lethal for several organisms unless *ung* (34), a gene present in *S. aureus*, is knocked-out. In this model, in order to bind to Stl, the phage encoded Duts should clean up this high cellular dUTP pool, then, in the absence of substrate, Dut would become available for interacting with Stl in a way that would not require the participation of motif V. Although the authors proposed that this alternative model dismissed our initial proposition of a G-like mechanism for the SaPIs induction by Duts, involving dUTP as a second messenger, we consider that their results conceptually confirmed our proposed signaling mechanism. Although contrary to our initial idea, the authors demonstrated that the dUTP-bound form blocks (instead of favors) SaPI induction. Indeed, their results clearly demonstrate that the dUTP is the second messenger that controls the on/off states of the Dut proteins in the Stl binding process.

**Figure 5.**
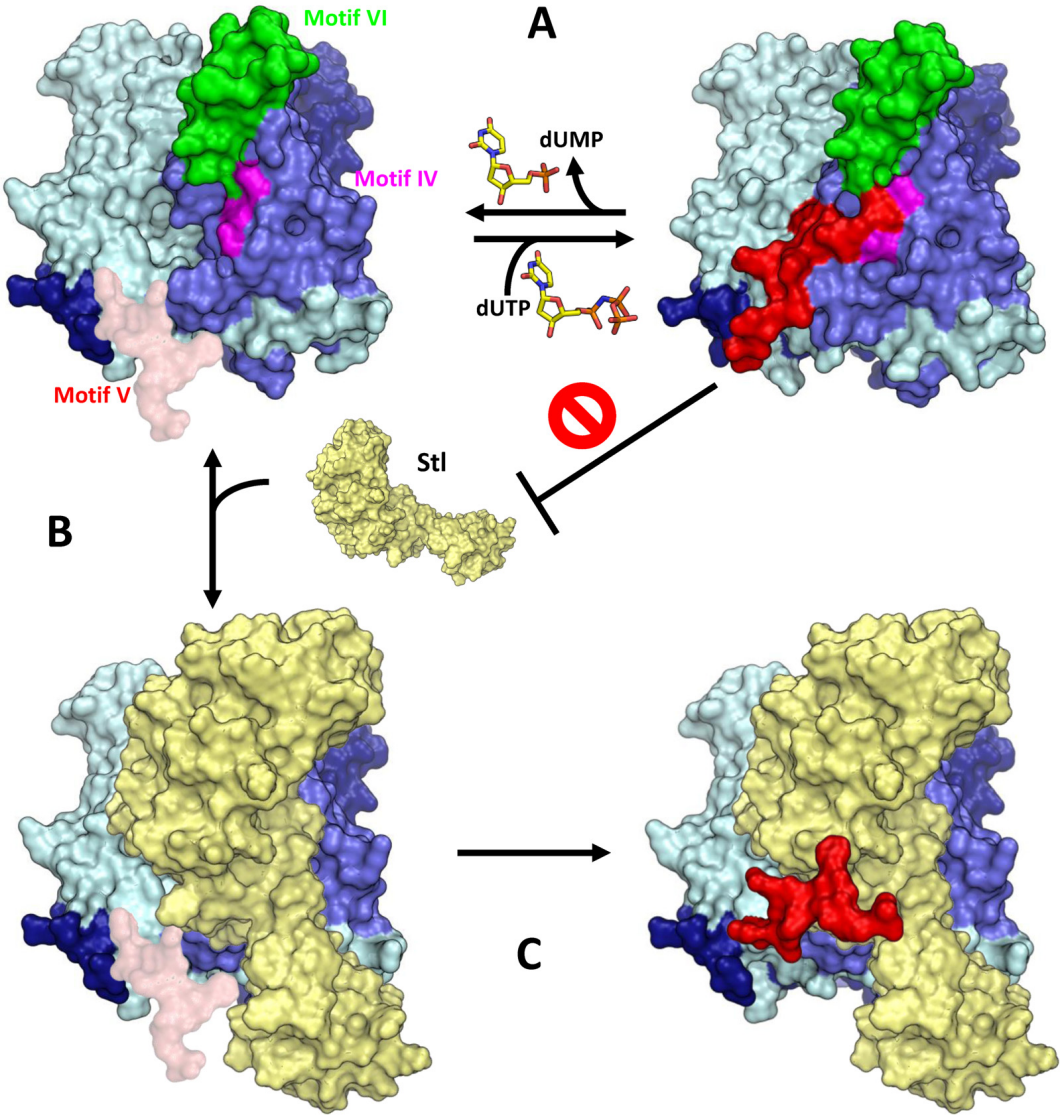
Model of *S. aureus* phage Dut interaction with SaPIs Stl. (**A**) Binding and hydrolysis of dUTP by Dut implicates the folding and stabilization of the high flexible conserved motif V (pink-red) over the active center. This disposition of motif V limited the access of Stl to their anchor place formed by motif IV (magenta) and motif VI (green) preventing the binding. (**B**) Contrary, in the Dut *apo* form, Stl anchor region is accessible due to the motif V flexibility, allowing Stl to interact with residues from motifs IV and VI in the vicinity of the active center. (**C**) Once Stl is bound, motif V would interact with Stl, stabilizing the complex. Structures are shown in surface using for Dut (diferent hues of blue) the experimental structures of Dut80α (PDB 3ZEZ) and for Stl (yellow) an *in silico* model generated by I-Tasser (39) from the Stl sequence. For clarity a single Stl is displayed, two additional Stl molecules following the tree-fold Dut symmetry should be present to complete the proposed 1:1 Dut–Stl interaction (13).

The inhibitory effect of dUTP on Stl:Dut binding, proposed by Szabo and co-workers, forced us to revisit our previous data in order to generate a model that could reconcile all the *in vitro* and *in vivo* observations. Since our previous structural and functional data clearly correlated the ordering of motif V with SaPI derepression, and given that motif V closing over the active site has been shown in the literature as the main conformational change induced by dUTP upon binding, it was surprising that this nucleotide precludes Stl:Dut interaction, blocking SaPI derepression. After confirming that dUTP also blocks the Stl:Dut80α interaction, we further analyzed the effect of the nucleotide on Stl:Dut interaction using some of the mutants previously characterized in our original study (9) and also generating a new battery of mutants that were identified in other complementary studies or specifically designed for this work. In parallel, we analyzed several of these mutations in Dutϕ11 to discard the possibility of two alternative mechanisms for Stl:Dut interaction. The *in vitro* and *in vivo* analyses of these mutants confirmed our previously proposed implication of motifs V and VI in Stl recognition and binding, but have brought to light the existence of differences among Duts in the contribution of each of these motifs in the Stl interaction process. In addition, this parallel analysis revealed that the conserved motif IV is also involved in Stl binding. This new uncovered interacting region maps where motifs V and VI meet and is involved in the nucleotide phosphate chain and in magnesium coordination (32). Indeed, the analyzed motif IV residues are in close vicinity of the pyrophosphate and C- terminal part of motif V, the two elements that show changes (the first is absent and the second alter its conformation), depending on whether it is the dUMP or the dUTP that is bound to the enzyme. This interesting result relates this interaction area with the differential effect of these two nucleotides in the Stl binding. Therefore, the parallel characterization has helped us to demarcate the Stl recognition area and to define the function of each motif: while the phage-specific motif VI and the Dut conserved motif IV should confer selectivity for Stl and would provide the initial recognition and main anchor point, the conserved motif V should work as a molecular switch hampering or stabilizing the Stl:Dut interaction. The switcher motif V would work in a nucleotide dependent way, with dUTP as second messenger, where the ‘off’ state corresponds to dUTP-induced conformation as was unveiled by Szabo and co-workers (13) and confirmed by us in the present work. In this way, we propose a revised model (Figure 5) where the Stl recognizes *apo* Duts by interacting with residues from motifs IV and VI in the vicinity of the active center. Once Stl is bound, the highly flexible motif V would interact with Stl, stabilising the complex. In this step, it is tempting to speculate that the Stl binding to Dut could work allosterically by promoting the motif V approach and interaction with Stl, mimicking the dUTP-induced motion of motif V. On the contrary, the dUTP binding to the *apo* Dut will induce the folding and stabilization of motif V over the active center, hampering the access of Stl to the anchor area and preventing the binding. Remarkably, this model is coherent with Szabo and co-workers’ observations and with our initially proposed model for Duts as signaling molecules with a mechanism conceptually analogous to G-proteins. Thus, the synergistic participation of three motifs in the Stl binding process, which show variability in sequence, explains the differences in the affinity for Stl observed between Dutϕ11 and Dut80α, accounting for the initial postulation of two alternatives models. Whist the combination of motif IV and VI in Dutϕ11 generates an Stl anchor area with extremely high affinity for Stl, in Dut80α this renders a low affinity binding site. In this scenario, the contribution of motif V to Dut:Stl complex stabilization is irrelevant for Dutϕ11 but crucial for Dut80α. Contrarily, motif V plays an identical role for both Duts in the dUTP-induced Stl binding inhibition, hampering Stl access to the anchor site.

The proposed model of Szabo and co-workers had additional physiological implications, attributing a sanitizing function to the phage Dut that provides a uracil-free replication environment for the SaPI. In the absence of experimental data concerning the effect of the *S. aureus* dUTP pool in SaPI biology, we found this proposition attractive but unfortunately our *in vivo* results with Dut defective phages do not support it. We consider that the Dut implication in the SaPI cycle is restricted to its induction. SaPIs are phage satellites that severely interfere with helper phage reproduction (38). Consequently, to avoid SaPI induction, phages evolve generating variants of the SaPI inducers with different affinities for the SaPI coded Stl repressors (29). With this strategy the phages try to encode variants of Dut proteins (Supplementary Figure S1) which could present lower affinity for the Stl repressor, but that are still functional for the phage. A very good example of this is reported here; the Dut80α has a significantly lower affinity for the Stl repressor than Dutϕ11. Remarkably, all the *S. aureus* phages encode Duts supporting the essentiality of this protein for the phage biology, not as an enzyme required to decrease the dUTP pool, as previously suggested, but by being involved in different stages of the phage reproduction cycle. Our recent results support the idea that the phage Duts have an important role in the phage biology (29). Taking advantage of the essentiality of this function, we propose that the Stl repressors have merged to mimic the structure of one of the partners with which the phage Duts interact, representing a fascinating example of molecular parasitism.

Finally, this proposition raises interesting questions about the molecular mechanism underlying the Stl:Dut interaction: if we assume that phages try to avoid SaPI induction by encoding Duts with low affinity for the Stl repressor, why does the ϕ11 encode a Dut with such high affinity for the Stl protein? Obviously, we do not have the answer to all of these questions yet, but we anticipate here that the Stl:Dut interaction will provide novel and unexpected answers about basic scientific questions, including what role these enzymes play in most organisms, from phage to humans.

## ACCESSION NUMBERS

Coordinates and structure factors have been deposited with the RCSB Protein Data Bank (http://www.rcsb.org/pdb/) under accession codes 5CCO and 5CCT for Dut80α^WT^-dUMP and Dut80α^G164S^-dUPNPP, respectively.

## Supplementary Material

Supplementary Data

SUPPLEMENTARY DATA
