# Supplementary material for: Another look at the mechanism involving trimeric dUTPases in Staphylococcus aureus pathogenicity island induction involves novel players in the party
Source: Nucleic Acids Res. 2016 Apr 25;44(11):5457–69. doi: 10.1093/nar/gkw317 (PMC4914113; doi:10.1093/nar/gkw317)
Supplement: SUPPLEMENTARY DATA [file supp_44_11_5457__index.html]

Another look at the mechanism involving trimeric dUTPases in Staphylococcus aureus pathogenicity island induction involves novel players in the party — Another look at the mechanism involving trimeric dUTPases in Staphylococcus aureus pathogenicity island induction involves novel players in the party — SUPPLEMENTARY DATA 

# Another look at the mechanism involving trimeric dUTPases in *Staphylococcus aureus* pathogenicity island induction involves novel players in the party

## SUPPLEMENTARY DATA

- SUPPLEMENTARY DATA
