# Supplementary material for: Another look at the mechanism involving trimeric dUTPases in Staphylococcus aureus pathogenicity island induction involves novel players in the party
Source: Nucleic Acids Res. 2016 Apr 25;44(11):5457–69. doi: 10.1093/nar/gkw317 (PMC4914113; doi:10.1093/nar/gkw317)
Supplement: SUPPLEMENTARY DATA [file supp_gkw317_gkw317-nar-01886-h-2015-File.zip › nar-01886-h-2015-File007.pdf]

Another look at the mechanism involving trimeric dUTPases in *Staphylococcus aureus* pathogenicity island induction involves novel players in the party.

Elisa Maiques, Nuria Quiles-Puchalt, Jorge Donderis, J Rafael Ciges, Christian Alite, Janine Bowring, Suzanne Humphrey, José R Penadés, Alberto Marina.

Table of Contents: Page

Supplementary Figures	2-6
Supplementary Tables	7-10
References	11

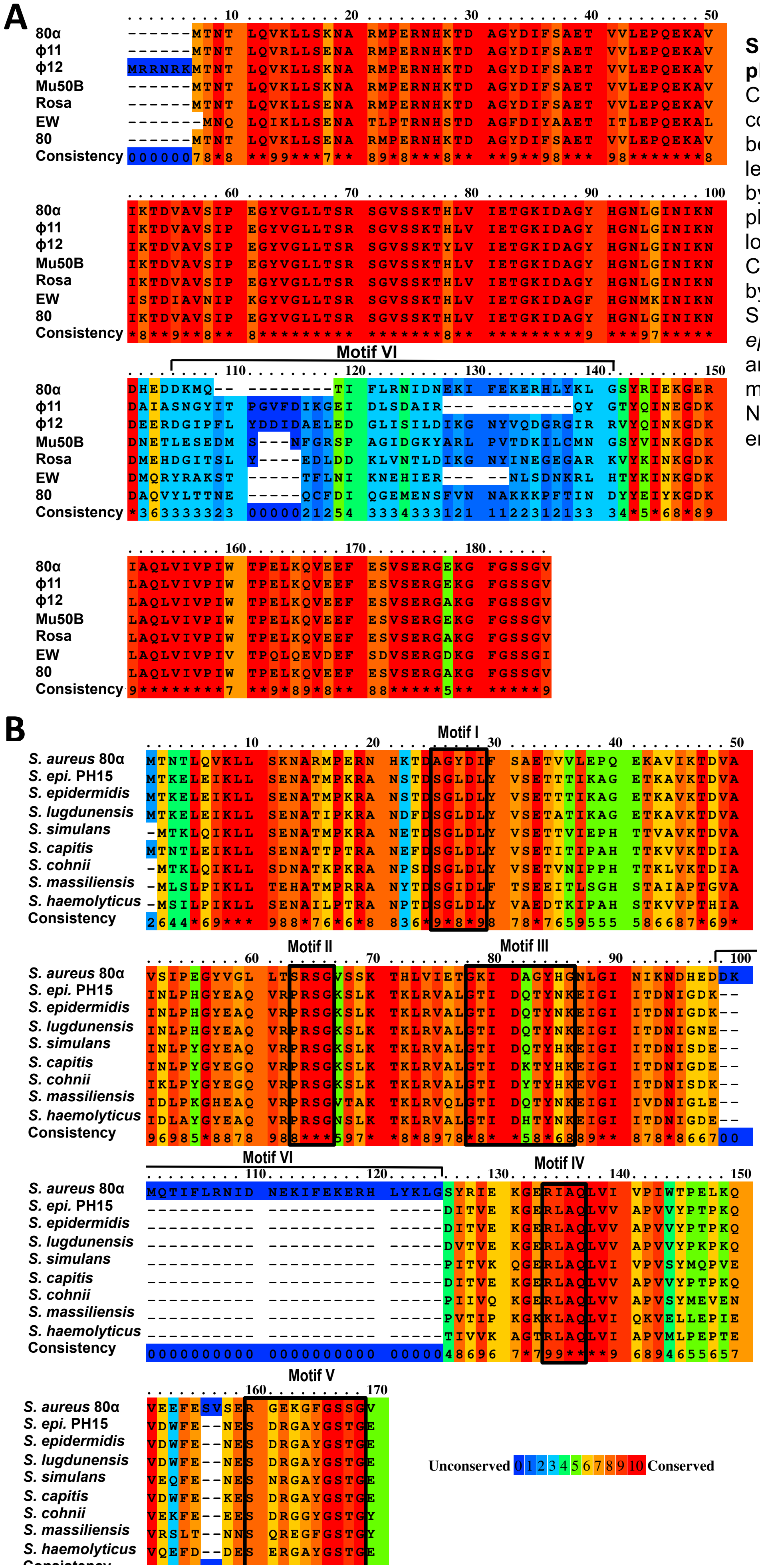

Supplementary Figure 1. Alignment of phage coded Dut protein sequences. Colours indicate relative sequence conservation at each position, with red being most conserved and blue being least (adapted from alignment generated by PRALINE). (A) Duts from *S. aureus* phages. The bracket indicates the localisation of the extra motif VI. (B). Comparison of the Dut proteins encoded by different Staphylococcal phages. The S. *aureus* phage 80α and the *S. epidermidis* phage PH15 are in the first and second position. The conserved Dut motifs I-V are highlighted and named. Note the absence of the motif VI in Dut encoded by not *S. aureus* phages.

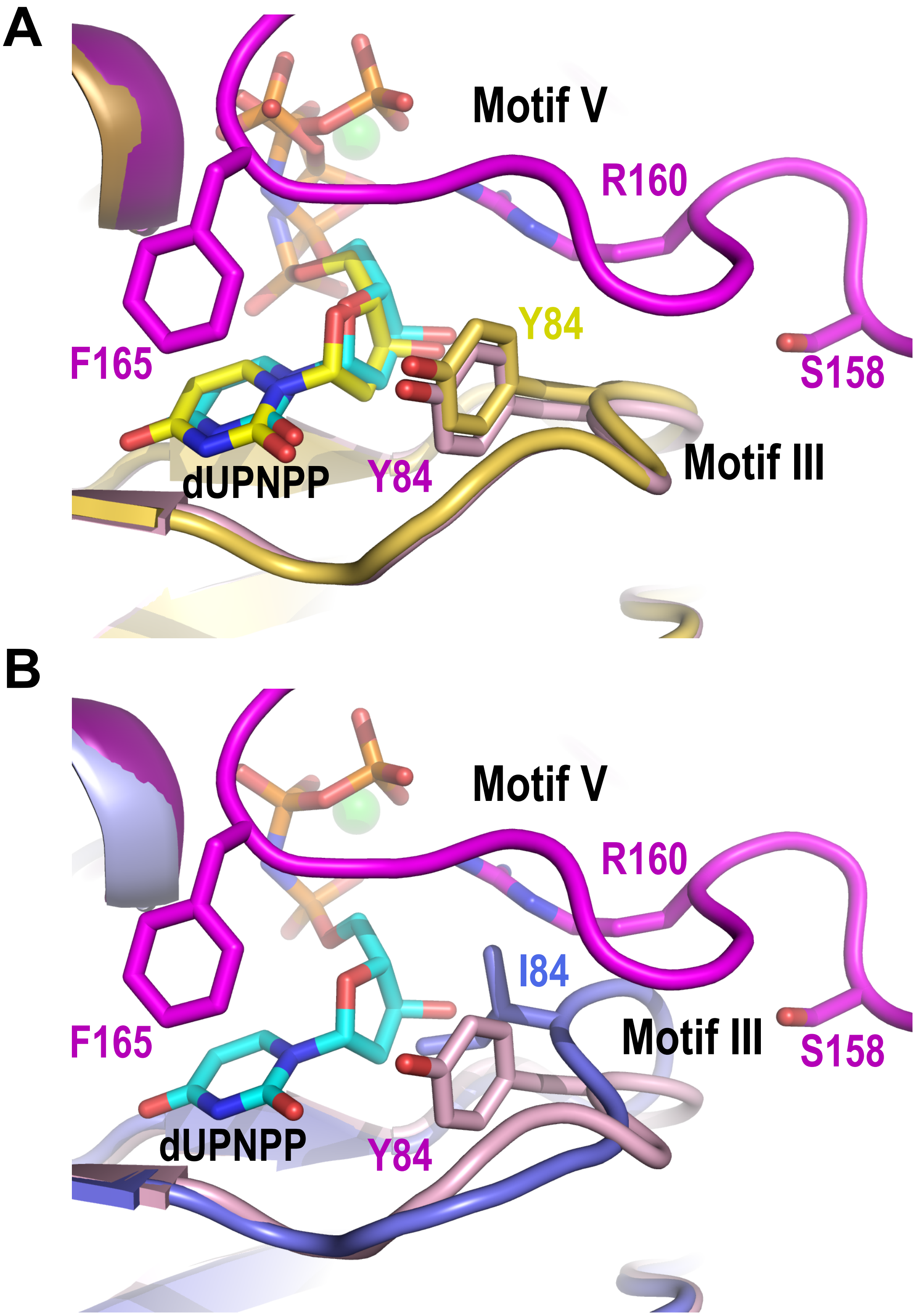

Supplementary Figure 2. Modelled C-terminal motif V in Dut80α^{D81A} and Dut80α^{Y84I} structures. Superposition of Dut80α^{WT} bound to dUPNPP (pink hues; PDB 3ZEZ) over the structures of (A) Dut80α^{D81A} (yellow hues; PDB 3ZF0) and (B) Dut80α^{Y84I} (blue hues; PDB 3ZF3) was used to place the C-terminal motif V (magenta) over the active center of these structures. dUPNPP from Dut80α^{WT} and Dut80α^{D81A} structures is shown in sticks with carbon atoms in cyan and yellow respectively. Relevant residues interacting with the nucleotide and/or motif III are shown in sticks with carbon atoms in the colour of the corresponding subunit and labelled.

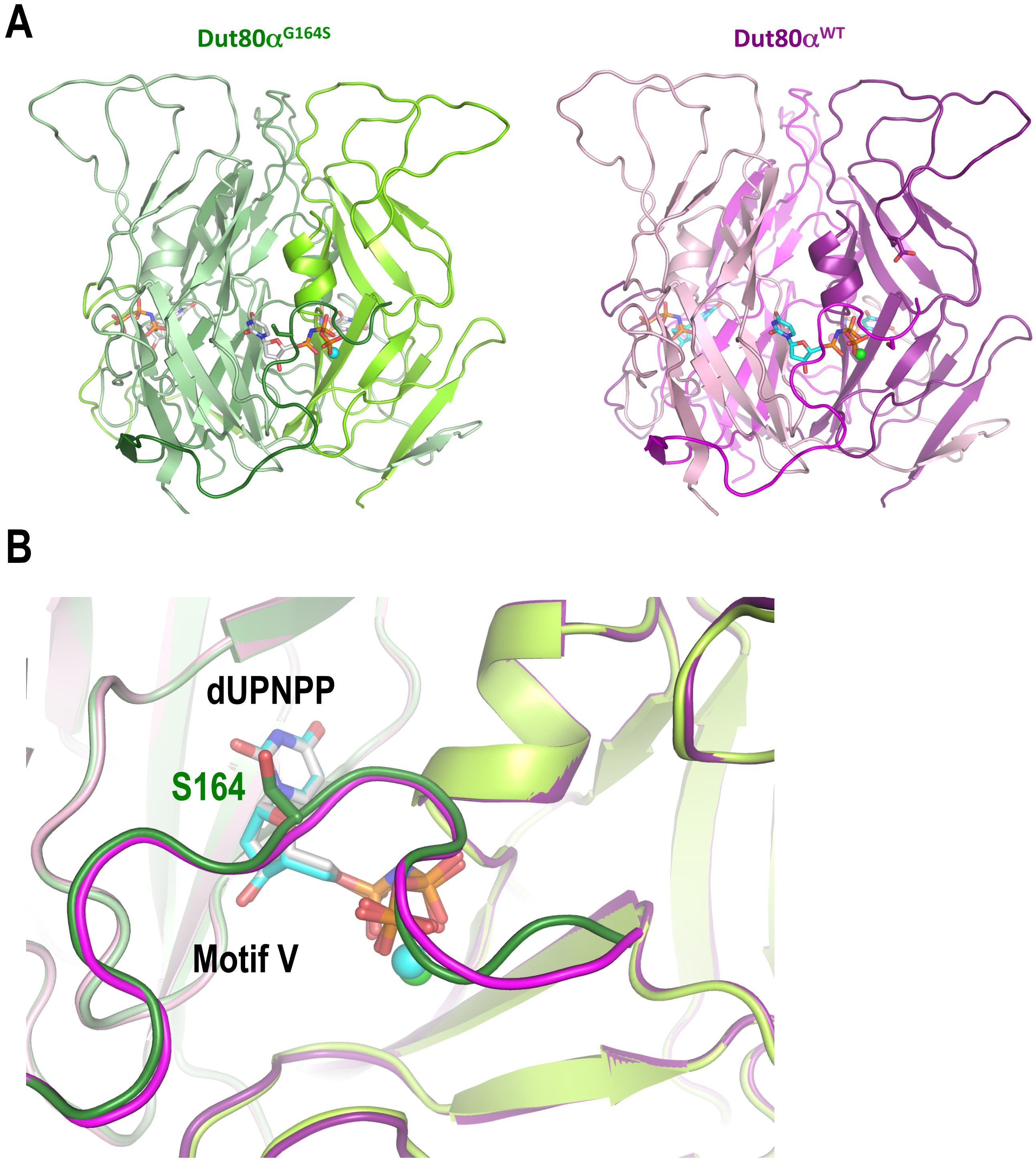

Supplementary Figure 3. Dut80α^{G164S} mutant structure and comparison with Dut80α^{WT}. (A) The crystal structure of Dut80α^{G164S} (left in green hues) bound to dUPNPP shows that this mutant presents an identical conformation that the wild-type protein bound to the same nucleotide (right, pink hues). (B) The superposition of both structures shows an almost identical way of nucleotide binding (in sticks) and C-terminal motif V conformation, distinguishing only for the solvent exposed disposition of the new Ser residue at position 164 (in sticks) in the mutant protein.

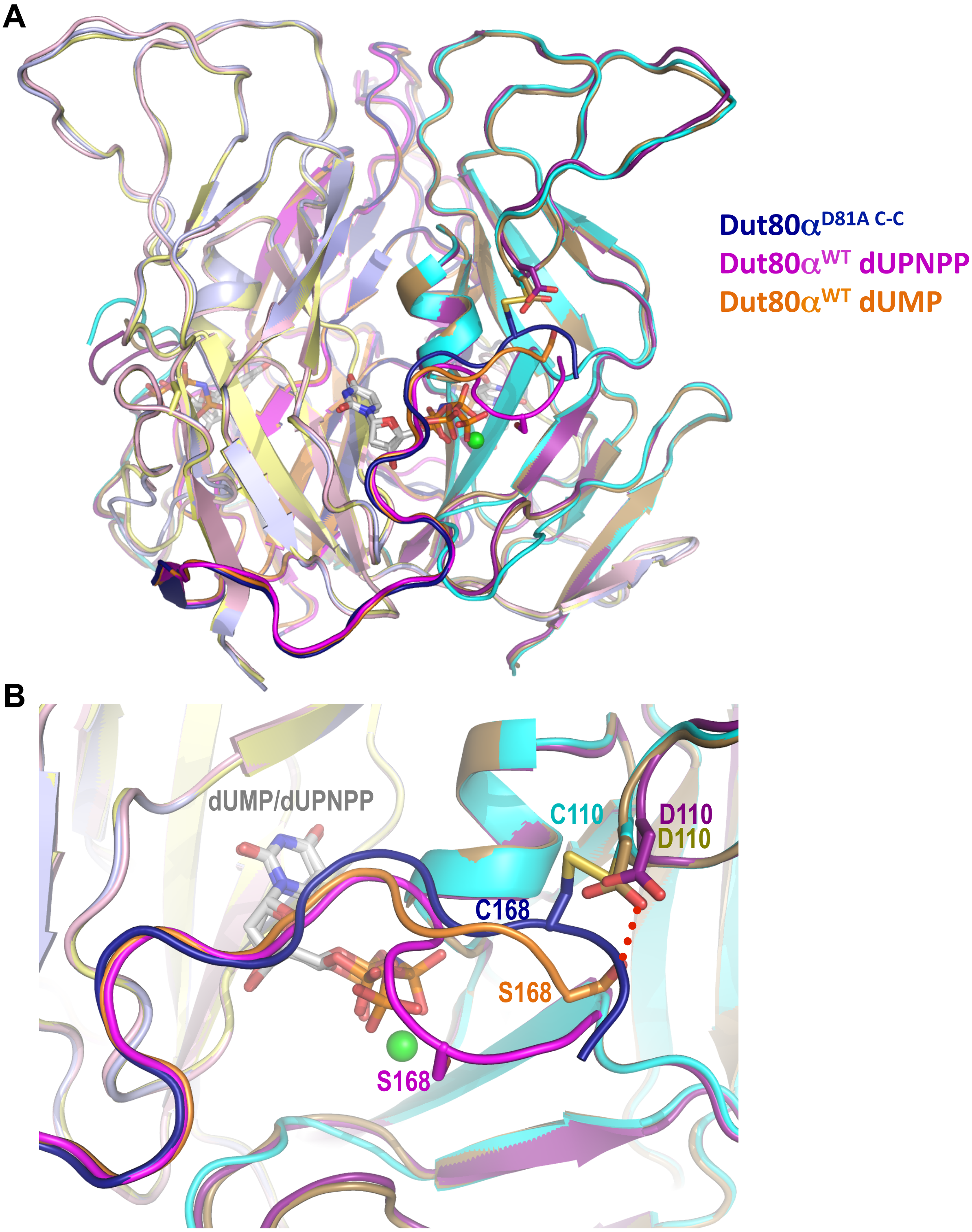

Supplementary Figure 4. Conformational changes induced by the nucleotide are restricted to the C-terminal motif V. (A) Superposition of the trimeric structures of Dut80α in the presence of dUPNPP (pink hues; PDB 3ZEZ) and dUMP (yellow hues) with the mutant Dut80α$^{D81A\ C\text{-}C}$ (blue hues; PDB 3ZF6) shows limited conformational changes that are restricted to C-terminal motif V. (B) A detailed view of the active centers of this structures shows that the four terminal residues of the motif V acquires alternative conformations in the presence of dUMP and dUPNPP placing in the former case these four residues to interaction distance of motif VI (hydrogen bond between D110 and S168 is highlighted by a dotted red line). The motif V conformation on the dUMP bound form is almost identical to the observed in the Dut80α$^{D81A\ C\text{-}C}$ mutant.

A

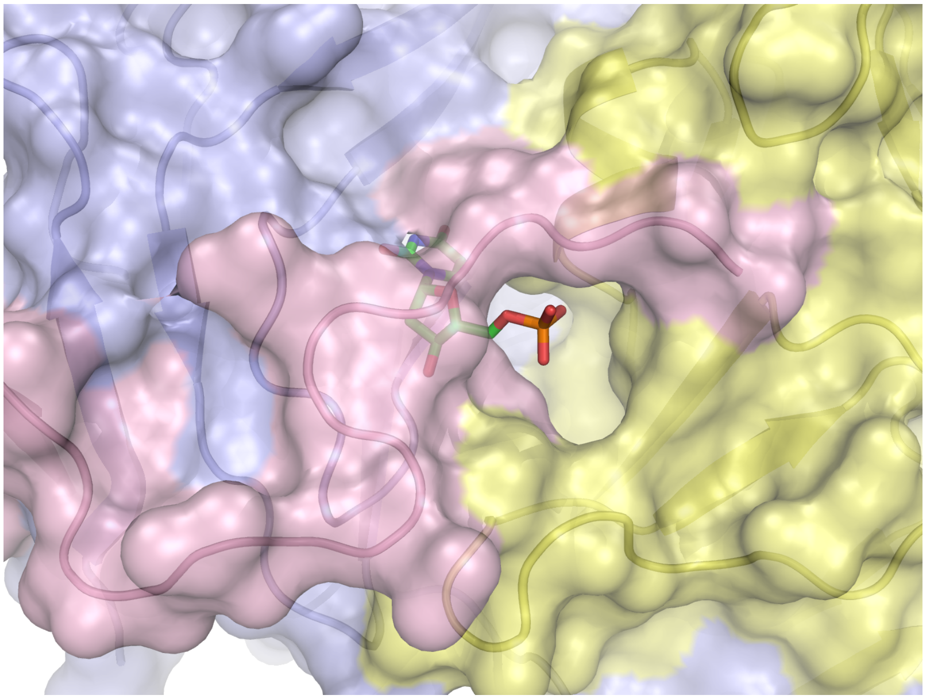

B

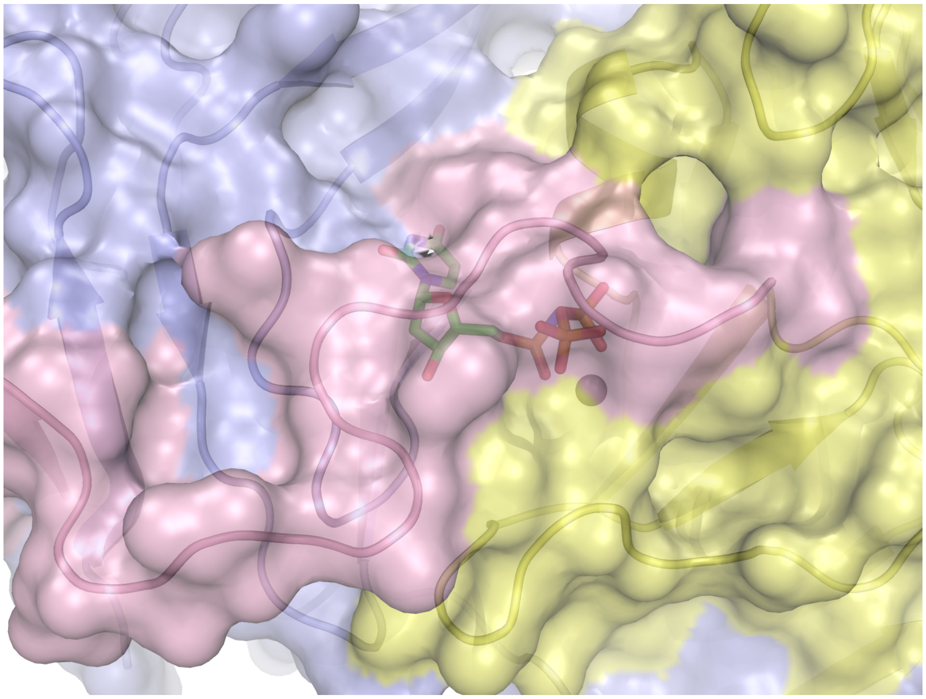

Supplementary Figure 5. Motif V conformation induced by dUMP opens the active center. Close view of the active center of Dut80α bound to (A) dUMP and (B) dUPNPP (PDB 3ZEZ) rendered in semi-transparent surface to show the bound nucleotide (in sticks with carbon atoms colored in green). Each subunit is shown in cartoon and colored in yellow, blue and pink respectively. Motif V corresponds to the subunit colored in pink. In the dUMP bound structure (A) the αP is solvent exposed by a channel that could be used to release the product pyrophosphate, while in the dUPNPP bound structure (B) the nucleotide is completely sealed

Table S1. Strains used in this study.

Strains	Description	Reference
RN4220	Restriction-defective derivative of RN450	(1)
RN10359	RN450 lysogenic for 80α	(2)
JP3603	RN10359 SaPIbov1 *tst*::*tet*M	(3)
JP6032	RN10359 Δ*dut*	(4)
JP6132	RN10359 Δ*dut* SaPIbov1 *tst*::*tet*M	(4)
JP12844	RN10359 SaPIbov1 *tst*::*tet*M Δ*stl*	This work
JP12845	RN10359 Δ*dut* SaPIbov1 *tst*::*tet*M Δ*stl*	This work
JP6774	RN4220 Δ*spa* SaPIbov1 *tst*::*tet*M	(4)
JP9026	JP6774 (pJP821)	(4)
JP12398	JP6774 (pJP1701)	This work
JP9032	JP6774 (pJP1159)	(5)
JP12834	JP6774 (pJP1703)	This work
JP12853	JP6774 (pJP1553)	This work
JP6789	JP6774 (pJP813)	(4)
JP13469	JP6774 (pJP1820)	This work
JP12397	JP6774 (pJP1789)	This work
JP13470	JP6774 (pJP1821)	This work
JP13472	JP6774 (pJP1823)	This work
JP13477	JP6774 (pJP1827)	This work
JP13478	JP6774 (pJP1840)	This work
JP13474	JP6774 (pJP1825)	This work
JP13475	JP6774 (pJP1839)	This work
DH5α	*E. coli* laboratory strain	Invitrogen
BL21(DE3)	*E. coli* expression strain	Stratagene
JP5359	BL21(DE3) (pJP753)	(4)
JP9351	BL21(DE3) (pJP1146)	(5)
JP8434	BL21(DE3) (pJP1147)	(5)
JP8474	BL21(DE3) (pJP1132)	(5)
JP9350	BL21(DE3) (pJP1145)	(5)
JP10636	BL21(DE3) (pJP1562)	(6)
JP12843	DH5α (pJP1704)	This work
JP12471	DH5α (pJP1702)	This work
JP13393	DH5α (pJP1838)	This work
JP13434	DH5α (pJP1843)	This work
JP4560	BL21(DE3) (pJP666)	(4)
JP13337	DH5α (pJP1828)	This work
JP12365	DH5α (pJP1795)	This work
JP13338	DH5α (pJP1829)	This work
JP13339	DH5α (pJP1830)	This work
JP13432	DH5α (pJP1841)	This work
JP13433	DH5α (pJP1842)	This work
AMC130707	BL21 (DE3) pETNKI-Stl	This work

Table S2. **Primers used in this study.**

Plasmid	Oligonucleotides	Sequence (5'-3')
pJP1701	orf32phi80alpha-16mS	ACGCGTCGACATTATGGCAGGTCAAGTTGTC
	dutphi80alpha-VI-2c	ACTGTCATTCTTGATATTAATCCC
	dutphi80alpha-VI-1m	GGGATTAATATCAAGAATGACAGTAACGGTAGTTACCGTATCGAAAAAGG
	orf32phi80alpha2cB	CGCGGATCCTCACCAAAACCTCCTTGACTC
pJP1703	orf32phi80alpha-16mS	ACGCGTCGACATTATGGCAGGTCAAGTTGTC
	dutphi80alpha-5cB	CGCGGATCCTCACCAAAACCTCCTTGACTCTATCTAATATGTCTTTACACTCCGCT ACTTCCTGCGCCTTTTTCTCCACGTTC
pJP1820	orf32phi80alpha-16mS	ACGCGTCGACATTATGGCAGGTCAAGTTGTC
pJP1821	dutphi11-10cB	CGCGGATCCTTAAACACTTTCGAATTCCTCC
		(these plasmids were constructed using the same oligonucleotides but different DNA templates)
pJP1789	orf32phi80alpha-16mS	ACGCGTCGACATTATGGCAGGTCAAGTTGTC
	dutphi80alpha-VI-2c	ACTGTCATTCTTGATATTAATCCC
	dutphi11-VI-1m	GGGATTAATATCAAGAATGACAGTAACGGAACTTATCAAATCAACG
	orf25phi11-5cB	CGCGGATCCCTTGACTCGATCTAAGATGTC
pJP1823	orf32phi80alpha-16mS	ACGCGTCGACATTATGGCAGGTCAAGTTGTC
	dutphi11-4cB	CGCGGATCCCCTTGACTCGATCTAAGATGTCTTTACACTCCGCTACTTCCTGCGCC TTTTTCTCCACGTTC
pJP1827	orf32phi80alpha-16mS	ACGCGTCGACATTATGGCAGGTCAAGTTGTC
pJP1840	dutphi80alpha-9c	CATATAGGTACAATAACTAATTGAGCTAGTTTGTCTCCTTTTTCGATACGGTAACT AC
	dutphi80alpha-8m	GTAGTTACCGTATCGAAAAAGGAGACAAACTAGCTCAATTAGTTATTGTACCTAT ATG
	orf32phi80alpha2cB	CGCGGATCCTCACCAAAACCTCCTTGACTC
		(these plasmids were constructed using the same oligonucleotides but different DNA templates)
pJP1825	orf32phi80alpha-16mS	ACGCGTCGACATTATGGCAGGTCAAGTTGTC
pJP1839	dutphi11-9c	CCATATAGGCACGATAACCAATTGAGCTATACGTTCTCCTTCGTTGATTTGATAA GTTCC
	dutphi11-8m	GGAACTTATCAAATCAACGAAGGAGAACGTATAGCTCAATTGGTTATCGTGCCT ATATGG
	orf25phi11-5cB	CGCGGATCCCTTGACTCGATCTAAGATGTC
		(these plasmids were constructed using the same oligonucleotides but different DNA templates)
pJP1702	orf32phi80alpha-12mB	CGCGGATCCATGACTAACACATTACAAGTAAAAC
pJP1704	orf32phi80alpha-13cS	ACGCGTCGACTCTTTACACTCCGCTACTTCC
pJP1838		(these plasmids were constructed using the same oligonucleotides but different DNA templates)
pJP1843		
pJP1795	orf25phi11-8mB	CGCGGATCCATGACTAACACATTACAAGTAAGG
pJP1841	orf25phi11-9cS	ACGCGTCGACCTTTACACTCCGCTACTTCCG
pJP1842		(these plasmids were constructed using the same oligonucleotides but different DNA templates)

Plasmid	Oligonucleotides	Sequence (5’-3’)
pJP1828	orf25phi11-8mB	CGCGGATCCATGACTAACACATTACAAGTAAGG
pJP1829	dutphi11-11cS	ACGCGTCGACTTAAACACTTTCGAATTCCTCC
		(these plasmids were constructed using the same oligonucleotides but different DNA templates)
pJP1830	orf25phi11-8mB	CGCGGATCCATGACTAACACATTACAAGTAAGG
	dutphi11-5cS	ACGCGTCGACCTTTACACTCCGCTACTTCCTGCGCCTTTTTCTCCACG
pETNKI-Stl	Stl-M1SUMO-FW	CCAGCAGCAGACGGGAGGTATGGAAGGAGCTGGTCAAATGGCAG
	Stl-N267 SUMO-RV	GGCGGCGGAGCCCGTTAATTAGTGTCTTTTTCAAGTATGATTTTTTTTTG

Southern blot	Oligonucleotides	Sequence (5’-3’)
SaPIbov1 probe	SaPIbov1-112mE	CCGGAATTCAATTGCTGAGGCAAAACTTC
	SaPIbov1-113cB	CGCGGATCCTAATTCTCCACGTCTAAAGC

Table S3. Plasmids used in this study.

Plasmid	Description	Reference
pCN51	Expresion vector	(7)
pJP821	pCN51-3xflag-*dut* 80α	(4)
pJP1701	pCN51-3xflag-*dut*$^{\Delta VI}$ 80α	This work
pJP1159	pCN51-3xflag-*dut*$^{\Delta V}$ 80α	(5)
pJP1703	pCN51-3xflag-*dut*^{F165A} 80α	This work
pJP1553	pCN51-3xflag-*dut*^{G164S} 80α	(6)
pJP813	pCN51-3xflag-*dut* 11	(4)
pJP1820	pCN51-3xflag-*dut*$^{\Delta V}$ 11	This work
pJP1789	pCN51-3xflag-*dut*$^{\Delta VI}$ 11	This work
pJP1821	pCN51-3xflag-*dut*$^{\Delta V\text{-}\Delta VI}$ 11	This work
pJP1823	pCN51-3xflag-*dut*$^{\Delta VI\text{-}F164A}$ 11	This work
pJP1827	pCN51-3xflag-*dut*$^{\Delta VI\text{-}IV11}$ 80α	This work
pJP1840	pCN51-3xflag-*dut*^{IV11} 80α	This work
pJP1825	pCN51-3xflag-*dut*$^{\Delta VI\text{-}IV80\alpha}$ 11	This work
pJP1839	pCN51-3xflag-*dut*$^{IV80\alpha}$ 11	This work
pET28a	Expresion vector	Novagen
pJP753	pET28a-*dut* 80α	(4)
pJP1146	pET28a-*dut*$^{\Delta V}$ 80α	(5)
pJP1147	pET28a-*dut*$^{D81A\ C\text{-}C}$ 80α	(5)
pJP1132	pET28a-*dut*^{D81A} 80α	(5)
pJP1145	pET28a-*dut*^{Y84I} 80α	(5)
pJP1562	pET28a-*dut*^{G164S} 80α	(6)
pJP1704	pET28a-*dut*^{F165A} 80α	This work
pJP1702	pET28a-*dut*$^{\Delta VI}$ 80α	This work
pJP1838	pET28a-*dut*$^{\Delta VI\text{-}IV11}$ 80α	This work
pJP1843	pET28a-*dut*^{IV11} 80α	This work
pJP666	pET28a-*dut* 11	(4)
pJP1828	pET28a-*dut*$^{\Delta V}$ 11	This work
pJP1795	pET28a-*dut*$^{\Delta VI}$ 11	This work
pJP1829	pET28a-*dut*$^{\Delta V\text{-}\Delta VI}$ 11	This work
pJP1830	pET28a-*dut*$^{\Delta VI\text{-}F164A}$ 11	This work
pJP1841	pET28a-*dut*$^{\Delta VI\text{-}IV80\alpha}$ 11	This work
pJP1842	pET28a-*dut*$^{IV80\alpha}$ 11	This work
pETNKI-1.10	Expression vector (hisSUMO3-LIC)	NKI Protein Facility
pETNKI-Stl	pETNKI-1.10-Stl	This work

References

1. Kreiswirth BN *et al*. (1983) The toxic shock syndrome exotoxin structural gene is not detectably transmitted by a prophage. *Nature.* 305(5936):709–712.
2. Ubeda C et al. (2007) A pathogenicity island replicon in *Staphylococcus aureus* replicates as an unstable plasmid. *Proc Natl Acad Sci USA.* 104(36):14182–14188.
3. Tormo MA et al. (2008) *Staphylococcus aureus* pathogenicity island DNA is packaged in particles composed of phage proteins. *J Bacteriol.* 190(7):2434–2440.
4. Tormo-Más MA et al. (2010) Moonlighting bacteriophage proteins derepress staphylococcal pathogenicity islands. *Nature.* 465(7299):779-782.
5. Tormo-Más MA et al. (2013) Phage dUTPases control transfer of virulence genes by a proto-oncogenic G protein-like mechanism. *Mol. Cell.* 49(5):947–958.
6. Frígols B et al. (2015) Virus satellites drive viral evolution and ecology. *PLoS Genet*. 11(10):e1005609.
7. Charpentier E et al. (2004) Novel cassette-based shuttle vector system for gram-positive bacteria. *Appl Environ Microbiol.* 70(10):6076–6085.
